# Integrating melt electrowriting (MEW) PCL scaffolds with fibroblast-laden hydrogel toward vascularized skin tissue engineering

**DOI:** 10.1016/j.mtbio.2025.101593

**Published:** 2025-02-19

**Authors:** Xixi Wu, Fenghua Zhao, Hui Wang, Romana Schirhagl, Małgorzata K. Włodarczyk-Biegun

**Affiliations:** a Department of Biomaterials and Biotechnology, University Medical Centre Groningen and University of Groningen, Ant. Deusinglaan 1, 9713 AV, Groningen, the Netherlands; b Nanostructured Materials and Interfaces, Zernike Institute for Advanced Materials, Faculty of Science and Engineering, University of Groningen, Nijenborgh 4, 9747, AG, the Netherlands; c Polymer Science, Zernike Institute for Advanced Materials, University of Groningen, Nijenborgh 4, 9747, AG, the Netherlands; d Biotechnology Centre, The Silesian University of Technology, Krzywoustego 8, 44-100, Gliwice, Poland

**Keywords:** Skin tissue engineering, Vascularized skin models, Scaffold design, PCL/GelMA composites, Skin mechanical properties

## Abstract

Three-dimensional (3D) skin equivalents (SEs) are promising platforms for studying skin disease or assessing the safety of skin-relevant products. Vascularization, which improves the functionality of reconstructed skin, is one of the remaining hurdles in SE production that, when successfully introduced, can widen SE applications. Here, combining porous polycaprolactone (PCL) melt electrowritten (MEW) scaffolds with fibroblast-laden methacrylated gelatin hydrogel (GelMA), we developed SEs with cellular vascular structure. The MEW scaffolds were composed of two layers: random fibers for culturing the keratinocytes to fabricate the epidermis; and well-aligned shapes filled with fibroblast-laden GelMA to mimic the dermis. Three dermal designs varying in porosities and pore sizes were compared to optimize the dermis reconstruction. Within one week, the design with bigger pore sizes achieved optimal cell distribution, penetration, and extracellular matrix (ECM) deposition. Additionally, Retinoic acid (RTA) was tested for improving ECM deposition. To mimic vasculature, we incorporated vascular grafts into the optimized SEs. These were fabricated by casting endothelial fibroblast-laden Matrigel onto small-diameter MEW-tubular structures. The versatility and reproducibility of the obtained SEs offer a robust new tool for *in vitro* testing and exploration of fundamental biological processes of skin tissue.

## Graphical abstract

The Figure: Human skin was generated by Biorender, the scientific illustration software (Canada), and the publishing certificate was obtained.

**Figure:**
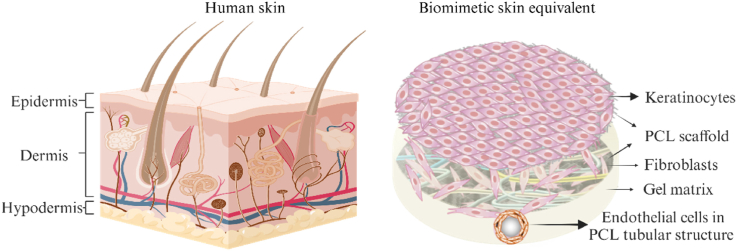

## 1 Introduction

Skin equivalents (SEs) that replicate the biological and mechanical properties of native tissue serve as promising platforms for evaluating skin products and exploring fundamental biological processes [1]. In the past decades, many efforts have been dedicated to fabricating skin models that accurately replicate the native skin structure and biological functions [[2], [3], [4]]. Compared to animal models, 3D biomimetic SEs containing human cells more accurately mimic properties of human skin. They also allow minimized individual variance and avoid ethical concerns [[5], [6], [7]].

Yet, accurate replication of a fully multilayered 3D structure of the skin is very challenging [8]. The natural skin tissue has a complex architecture with an interplay of different morphological, biochemical, and physiological properties [9]. It is composed of the epidermis, dermis, and subcutaneous layer with vasculature beneath them [10,11]. At present, 3D-engineered skin constructs containing both epidermis and dermis layers have been developed. They were fabricated using cellular hydrogel, cellular scaffolds composed of natural-based materials (like collagen, and elastin separately or combined) [12,13] or polymeric materials (Poly(caprolactone) (PCL), poly(ethene glycol) (PEG), poly(vinyl alcohol) (PVA), poly(lactic acid) (PLA) and polystyrene seeded with cells [[14], [15], [16], [17], [18]], or using the three components simultaneously (hydrogel, polymeric scaffolds, and cells) [[19], [20], [21]].

The most common hydrogel skin models containing cells include keratinocytes and human dermal fibroblasts, serving as versatile and powerful platforms for drug testing and skin products evaluation [[22], [23], [24], [25]]. The main aim of engineering these structures is to mimic skin biological properties. They have good biocompatibility and cellular behavior including adhesion, spreading, proliferation, and migration. However, these hydrogel models are weak in mechanical properties, which impede resistance to external forces [26], affect the homeostasis of skin healing, and slow skin reorganization [27,28]. While there are several skin models where the mechanical properties have been taken into account [1,12], this is still a relatively new area of research. One of the approaches to achieving such improved skin models is using porous dried polymer scaﬀolds [29]. They have comparable advantages to bulk hydrogel models, with tunable mechanical strength. Several fabrication methods of scaffolds have been proposed, including freeze-drying [[30], [31], [32], [33]], 3D printing [[34], [35], [36], [37]], and electrospinning [[38], [39], [40], [41]].

Electrospinning is an interesting and widely used technique for tissue regeneration (especially skin models). It enables the fabrication of a highly porous and interconnected fibrillar framework [42] resembling the organization of ECM or collagen fibers. However, a major limitation of electrospinning is the limited control over material deposition and, therefore, the final scaffold architecture. Furthermore, it allows to produce only flat and typically very dense scaffolds. As a result, cell penetration in 3D is hampered. A related technique allowing for the controlled deposition of fine fibers into three-dimensional scaffolds with increased and fully controlled porosity is melt electrowriting (MEW).

MEW is an emerging biofabrication method that combines the precision of 3D printing with the principles of electrospinning. It utilizes high voltage to deposit molten polymer material accurately, resulting in fibers typically in the micrometer range [43]. The most commonly used polymer for MEW, PCL, is FDA-approved and widely regarded as the gold-standard material due to its ease of processing and exceptional printability [44,45]. PCL scaffolds have good biocompatibility and long-term biodegradability [1,46]. The flexibly adjustable 3D structural design and precise fiber placement ensure tailored topology and the formation of volumetric structures with the space available for cells to penetrate between the fibers. The mechanical features of the MEW structures can be customized, and therefore provide cues controlling cell behavior and fitting to the targeted tissue. To further improve the specific cellular response and accuracy of the model, MEW scaffolds may be integrated with the hydrogel matrix.

Composite systems based on hydrogel and fibrous reinforcement are attractive scaffolds for 3D tissue culture *in vitro*, due to the resemblance to native ECM and outstanding flexibility in design and functional properties [47]. The use of cellular hydrogel keeps cells in the porous scaffold and forms ECM quickly, whereas the customized polyester scaffolds ease the handling of the artificial tissue and increase its mechanical properties. Visser et al. [48] first showed that the compressive modulus of MEW printed PCL/GelMA composites was increased up to 54-fold when compared to GelMA or PCL scaffold alone. Castilho et al. [49] revealed the reinforcement mechanism in the compression of the composite constructs. The effect was assigned to the fibers in PCL scaffolds, pulling against the tension caused by the lateral expansion of the hydrogel. Additionally, the gel hindered the buckling of the scaffold. The compression modulus of GelMA hydrogel/MEW scaffold composite was much higher than of pure MEW scaffolds, i.e., E = 387 ± 34.6 kPa vs E = 14.1 ± 1.9 kPa, because the hydrogel provided resistance against deformation of the scaffold. These studies revealed the great impact of embedding fibrous scaffolds obtained with MEW in a hydrogel matrix.

Several attempts to use MEW in skin tissue engineering were reported. Hewitt et al. [50] MEW-printed milk protein/PCL scaffolds to fabricate skin bilayers with fibroblasts and keratinocytes. However, the distribution of the fibroblasts and the infiltration of keratinocytes into the dermis layer were hard to control due to the big pore sizes in the scaffolds (over 200 μm). Additionally, the authors did not investigate the mechanical properties of the obtained skin model. In another study, Ferdows et al. [51] proposed acellular hybrid scaffolds for wound healing, comprising a gelatin matrix, with growth factors, reinforced with MEW-printed PCL or PCL/bioactive glass meshes. A systematic study on the influence of PCL fiber design on skin regeneration was recently conducted by Fabien et al. [1] The authors fabricated a skin structure by combining an electrospun membrane as the epidermal layer and the MEW-dermal compartment. They found that the fiber design of PCL scaffolds affected the organization of dermal ECM. The authors also tried to closely mimic the compressive modulus of the native dermis by adding cells and vitamin C to the scaffolds. Still, the model was missing vascularization which enhances nutrient and oxygen delivery in engineered skin tissue, while also improving its physiological relevance [52,53]. Additionally, inclusion of vasculature, allows to obtain more volumetric and viable skin constructs, facilitating scaling up and obtaining engineered construct of medically relevant size [54,55]. The vascularization would push the research on SEs forward. Further, there is still a need for SEs that closely mimic both, the compressive and tensile, moduli of native skin.

**Fig. 1:**
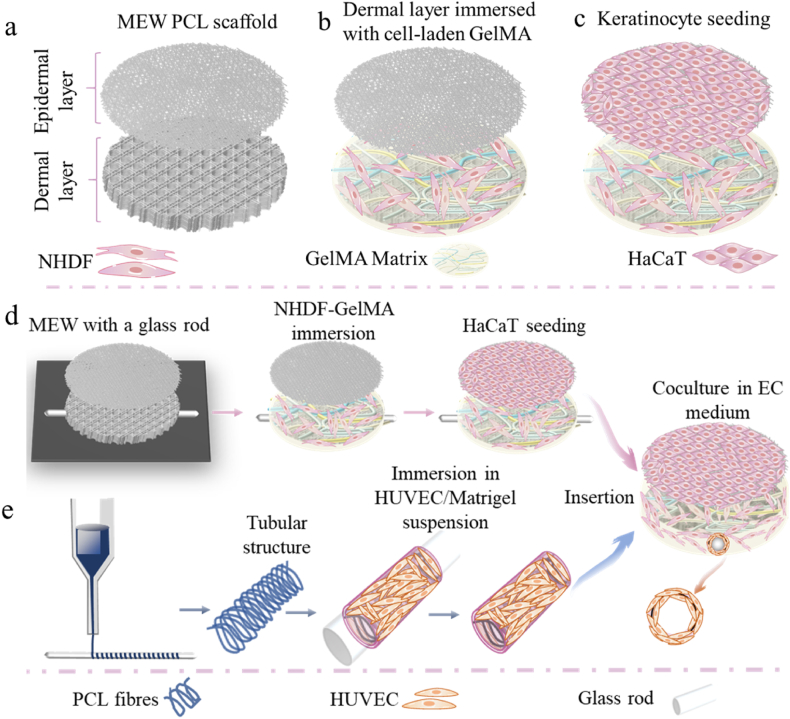
a-c) Fabrication process of SEs by combining PCL scaffolds with fibroblast-laden GelMA. a) Design and printing of PCL scaffolds. b) Generation of dermal compartment: immersing dermal layer in fibroblast/GelMA suspension and crosslinking the composite using UV irradiation, c) followed by keratinocytes seeding on a dense layer of randomly oriented fibers. d, e) Fabrication process of SEs with cellular tubular structure. d) Scaffold printing with the help of a 2 mm glass rod, and the preparation of SEs with the rod. e) Printing of tubular structure and the combination of HUVEC/Matrigel layer.

In this project, we developed SEs, forming within 10 days, by combining a PCL scaffold with cellular GelMA. To achieve this aim, multiple-layered PCL scaffolds were printed using MEW (Fig. 1a). PCL scaffolds served as structure-forming units and strengthening elements. Gelatin include the inferior mechanical stability of printed scaffolds, structural instability at physiological temperatures, and rapid dissolution in culture media during tissue culture. GelMA, which is a gelatin derivative modified with methacryloyl anhydride (MAAnh) that contains extracellular matrix (ECM)-like components, features RGD sequences (Arg-Gly-Asp peptides), which promote cell adhesion, spreading, and differentiation [56,57]. Furthermore, methacrylation enhances stability after UV crosslinking, a strategy commonly applied to other biomaterials such as silk, hyaluronic acid, and chitosan [[58], [59], [60]]. Compared to gelatin which has inferior mechanical properties, structural instability, and rapid dissolution in culture media during tissue culture, GelMA offers tunability, ease of use, customization, stability, and consistency [56], making it a highly advantageous choice for creating SEs compared to other hydrogels like collagen and dECM. GelMA containing normal human dermal fibroblasts (NHDF) was introduced into the scaffold part mimicking the dermis, followed by seeding keratinocytes on the top layer, composed of randomly distributed PCL fibers, to mimic the epidermis. The filling of voids in the MEW mesh by fibroblast-laden GelMA in the dermis layer, shortened the time needed for ECM reconstruction by seeded fibroblasts, while preventing the infiltration of keratinocytes into this layer from the epidermal zone. Random fibers helped form a waved epidermis that closely resembled the native skin structure. In the optimization process, three designs of dermal layers were compared to show the influence of scaffold architecture and porosity on cell penetration and distribution. Histological staining and secretion of relevant biomarkers of the optimized SEs were analyzed. The mechanical properties including compressive and tensile moduli of the obtained SEs were measured. Further, the efficacy of Retinoic acid (RTA) was evaluated based on the SEs. Finally, the vascular structure, with endothelial cells embedded in Matrigel, which worked better compared to GelMA here, was integrated into the existing SEs. The inclusion of the cellular tubular structure paves a new way for developing vascularized SEs, potentially advancing the applications of research in SEs. The proposed SEs have a great potential for toxicity testing, or exploration of fundamental biological processes of skin tissue.

## 2 Materials and methods

### 2.1 Fabrication of 3D PCL scaffolds using MEW method

**Fig. 2:**
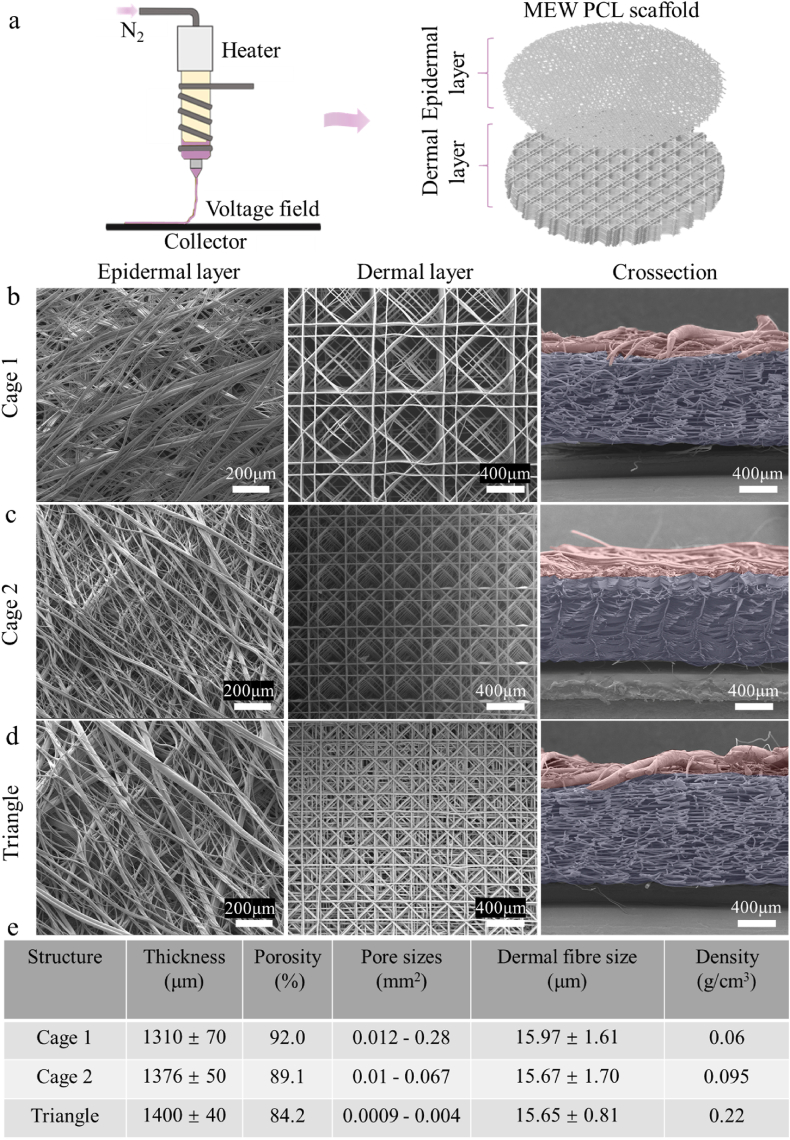
a) Schematics of a PCL scaffold printed by MEW. b-d) The SEM (epidermal, dermal, and crossection) images of Cage 1, 2, and Triangle (after NaOH etching). The SEM images were post-processed with Adobe Photoshop (the USA), in the crossection column, epidermal layers were labelled in pink, and the dermal layers were labelled in light purple. e) Summary of scaffold thickness, porosity, pore sizes, dermal fibre size, and density. 3 samples of each design were measured. (For interpretation of the references to color in this figure legend, the reader is referred to the Web version of this article.)

Polycaprolactone (PCL, Purasorb PC 12, Corbion) pellets were used for MEW. 2g of pristine PCL was transferred into a steel cartridge fitted with a 0.25 mm bronze nozzle (E3D online, UK) and loaded into the heating cylinder of the MEW printer (Spraybase® A-1204-0001-01D, Ireland). The printing temperature was set to 100 °C following the previous study [44]. The distance between the nozzle and the collector was kept constant at 5 mm, and the voltage at the level of 6 kV was used. Three designs for the dermal layers were printed, named Cage 1, Cage 2, and Triangle according to their respective final shapes. Cage 1 and Cage 2 share the same basic square design structure but differ in pore sizes: Cage 1 has pores ranging from 0.012 mm^2^ to 0.28 mm^2^, Cage 2 has smaller pores, from 0.01 mm^2^ to 0.067 mm^2^. Triangle design was characterized by 0.0009–0.004 mm^2^ pore sizes. The dermal layers had ca. 1 mm thickness (see Fig. 2b–d). The epidermal layers were composed of densely MEW-deposited random fibers. The printing pressure of the dermal layer was at 0.2 bar, and the printing velocity was 250 mm/min. The printing pressure used for obtaining the epidermal layer was 0.5 bar, and the velocity was 500 mm/min, which followed our previous research [61]. The final dimension of the printed scaffold was ca. 20 mm × 20 mm x 1.1 mm.

After fabrication, the morphology and cross-section of PCL scaffolds were analyzed by SEM (FEI Quanta 400 FEG, USA). Additionally, the liquid displacement technique was used to measure the density (g/cm^3^) of the scaffolds according to the methods described in a previous study [62]. PBS was used as displacement liquid. The weight of scaffolds (W) was measured and the material was dipped into a known volume (V1) of PBS in a measuring cylinder for 5 min. The total volume of PBS and PBS-impregnated scaffold was recorded as V2. Subsequently, the scaffold was removed from the measuring cylinder, and the remaining volume of PBS in the measuring cylinder was recorded as V3. By using the equations below, the density and porosity of the scaffolds were estimated.

(1) The total volume of the scaffold: V2

(2) The density of the scaffold: D = W/(V2 – V3)

(3) The porosity of the scaffold: P = (V1 – V3)/(V2 – V3)

### 2.2 Synthesis of GelMA

To prepare GelMA, the general protocol as reported by Van Den Bulcke et al. was used [63], with some minor modifications [[64], [65], [66], [67]]. In brief, 5 g Type A porcine skin gelatin (G1890, Sigma) was dissolved in 100 mL Dulbecco's phosphate buffered saline (DPBS, PH = 7.4, Sigma) for 1 h at 50 °C under vigorous stirring. Next, 4 mL methacrylic anhydride (MA, Sigma) was added dropwise into the gelatin solution. The reaction was left for 3 h followed by the dilution of the mixture with 100 mL DPBS. After 2 h, the blend was subjected to centrifugation to eliminate some unreacted species. The supernatant was then collected for dialysis using a 12–14 kDa dialysis bag (Sigma) in warm deionized water (40 °C). After one week of dialysis, the GelMA solution was lyophilized for 5–7 days to produce a porous white foam.

### 2.3 Characterization of GelMA

#### 2.3.1 Fourier transform infrared spectroscopy (FTIR) and NMR measurements

To confirm the chemical structure of GelMA, FTIR and 1H NMR characterizations were performed. A transmittance spectrum of GelMA foam was collected by an FTIR spectrometer (FTIR, Bruker IFS88, USA). The spectrum was measured in the wavelength range from 400 cm^−1^ to 4000 cm^−1^ and we conducted 64 scans. For 1H NMR analysis, gelatin and GelMA were dissolved in a D_2_O (Sigma) solution, and 1H NMR (Bruker Ascend 600 FT-NMR, USA) was utilized to determine the methacrylation of GelMA.

#### 2.3.2 Preparation of GelMA hydrogel

The GelMA solution was prepared following the protocols reported before [68]. In short, freeze-dried GelMA foam was dissolved in DPBS (5 % (w/v)) with 0.25 % (w/v) photoinitiator (Lithium phenyl-2,4,6-trimethylbenzoylphosphinate (LAP), Sigma) at 40 °C under vigorous stirring, these concentrations were used for the following studies.

##### 2.3.2.1 Compressive moduli of crosslinked GelMA and Matrigel

Compressive measurements of the crosslinked GelMA and Matrigel were performed with a customized low-load compression tester (LLCT, the Netherlands). The stiffness of 5 % (w/v) GelMA after different UV curing times: 5–60s was measured. UV irradiation was performed at a height of 10 cm above the samples, and the compression moduli were measured every 5s. 300 μL GelMA solution (the thickness was ca. 1 mm) with LAP was crosslinked homogenously in a petri dish by UV irradiation (405 nm, Amazon, the USA). The compressive moduli of 300 μL Matrigel at 50 v/v %, 75 v/v %, and 100 v/v % were also measured after 15-min thermal-crosslinking in a 37 °C incubator. The components of the LLCT instrument have been previously described in detail [53]. In this setup, the LabVIEW 7.1 program was employed to acquire data from the LLCT load cell and linear positioning system. The system achieved a resolution of 0.1 mm, 2 mg in load, and 25 ms in time. Motion velocity was controlled using feedback mode. The top plate moved downward (5 μm/s) until it experienced a counterforce of 10−4 N. Samples were deformed by 20 % of their original thickness (strain *ε* = 0.2) at a deformation speed of 2 %/s (strain rate $\varepsilon^{∙}$ = 0.2 s−1). The indentation probe had a diameter of 2.5 mm. The deformation was maintained at a constant level for 100 s, during which the applied stress was continuously monitored. During the compression process, a plot was generated depicting the relationship between the measured stress and the applied strain. A linear relationship between stress and strain was observed in the plot between a strain of 0.05 and 0.1. The slope of this line, representing stiffness (Young's modulus), was determined and the average of 3 samples was calculated.

##### 2.3.2.2 Cell performance in hydrogels

**Fig. 3:**
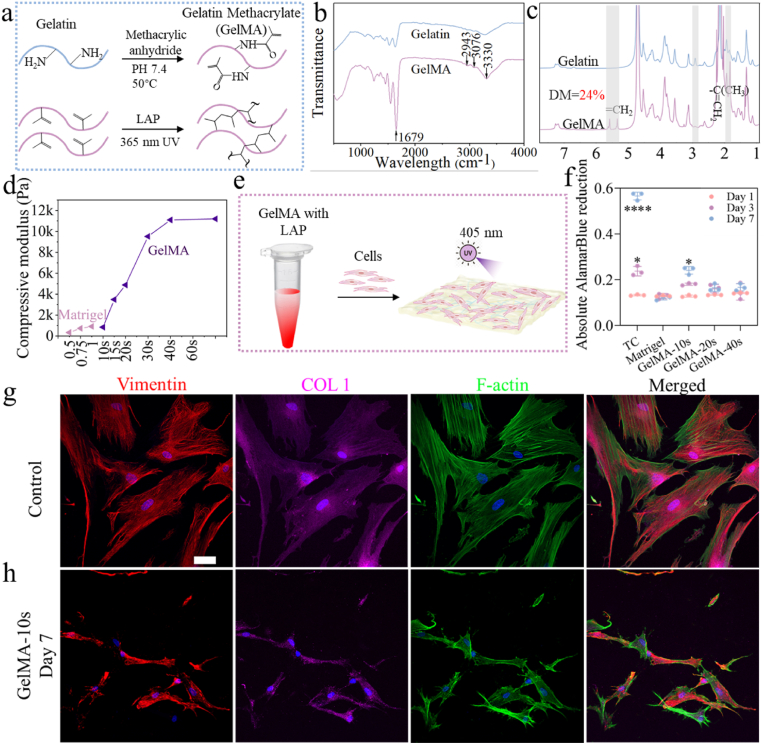
GelMA analysis. a) The introduction of methacrylation substitutions to form GelMA, and GelMA crosslinking in the presence of a LAP photoinitiator and UV irradiation. b) FTIR spectra of gelatin and GelMA, as well as peaks characteristic for methacrylation, are marked. c) 1H NMR spectrum of gelatin or GelMA. d) Compressive moduli of Matrigel (50 v/v. %, 75 v/v. %, and 100 v/v. %) and GelMA sampels after UV-crosslinking for 10s, 15s, 20s, 30s, 40 s nd 60s. e) The preparation procedure of cell/GelMA suspension and the following crosslinking. f) Cell metabolism in Matrigel (100 v/v. %), and GelMA crosslinked for 10 s, 20 s, and 40 s, after 1 day, 3 days, and 7 days culture. The data are shown as mean ± SD. *p* value ∗≤ 0.05, and ∗∗∗∗≤ 0.0001, one-way ANOVA tests, n: 3. Cells on Petri dishes were taken as the control group. Cell growth on g) Petri dishes, and in h) GelMA (10-s UV crosslinking) after 1-week culture. Vimentin (red), Collagen I, F-actin (green), and cell nuclei (blue) were stained to show morphology and migration capacity, Collagen I secretion, and spreading of cells, respectively. Scale bar: 50 μm. (For interpretation of the references to color in this figure legend, the reader is referred to the Web version of this article.)

The stiffness of 100 (v/v) % Matrigel was found to be similar to that of GelMA crosslinked for 10 s (Fig. 3d). To evaluate cell behavior in gels with similar compressive moduli, cell performance was compared between GelMA crosslinked at 10 s and 100 (v/v) % Matrigel. NHDF cells were cultured with cell medium containing 10 % FBS, 89 % DMEM, and 1 % penicillin-streptomycin in the 37 °C incubator with 5 % CO_2_. After reaching 70–80 % confluency, the cells were detached from the flasks using 0.25 % trypsin–EDTA, and the total number of cells was counted followed by centrifugation. GelMA with LAP solution was sterilized using 0.25 μm filters in a sterile laminar flow hood. A cell suspension at a concentration of 5.5 x 10^5^ cells/mL was obtained after homogeneously resuspending the cell pellet in GelMA with LAP solution, or Matrigel. Three replicates of fibroblast-laden samples (300 μL) were prepared in 4-chamber petri dishes. After irradiating with UV light (405 nm) at 10 cm above the samples for 10 s, 20 s, and 40 s, the GelMA samples were washed 3 times using DPBS, and 0.5 mL fresh cell medium was introduced to each chamber. 0.5 mL fresh cell medium was added to the Matrigel samples after they were crosslinked. The cell performance in GelMA crosslinked for 10 s was superior to that in 100 v/v % Matrigel (in Discussion **3.2.3.**). Based on these findings and following the same procedures mentioned in Section 2.3.2.2, we further investigated cell performance in GelMA crosslinked for 20 s and 40 s. This study aimed to examine how variations in stiffness and the time of UV irradiation affect cell behavior in GelMA.

To determine the cytotoxicity in GelMA, Fluorescein Diacetate/Propidium Iodide (FDA/PI, Sigma) was applied to test the cell survival rates after 7-day cell culture. Briefly, 200 μL DPBS solution containing 6 μg/mL FDA and 20 μg/mL PI was added to a single chamber after removing the cell medium, and incubated for 15 min at room temperature (RT). The stained samples were washed 3 times using DPBS and visualized using a Zeiss confocal microscope (Zeiss 710, Germany), and images were analyzed using ImageJ (the USA). The result is presented in Figure SI 1. The cell metabolism at days 1, 3, and 7 was measured by an AlarmaBlue kit following our previous study [69].

Additionally, immunostaining was performed to analyze the cytoskeleton and the expression of vimentin, a marker for fibroblastic proliferation, and type I collagen, a marker for ECM deposition, after 1 week of culture. The GelMA and Matrigel samples were washed with DPBS followed by fixation with 4 % paraformaldehyde (PFA) for 10 min. Next, the samples were rinsed with DPBS three times and treated with immunostaining permeabilization solution (Triton 100x, 0.05 %) for 10 min. After washing three times with DPBS, 5 % BSA buffer was applied for 30 min to prevent non-specific protein binding on cell membranes. Then BSA was removed and Mouse Anti-Vimentin antibody (1:200 in DPBS, Sigma) and Rabbit Anti-Collagen I antibody (1:500 in DPBS, Fisher Scientific) were added to the samples. Incubation was performed at 4 °C overnight. Afterward, samples were washed with DPBS, and then incubated with the corresponding secondary antibodies for 45 min at room temperature (RT). Excess of antibodies was removed by three washes with PBS and cell microfilaments were stained by FITC labelled Actin-Tracker for 20 min. Then, the samples were washed three times with DPBS. 2-(4-amidinophenyl)-6-indolecarbamidine dihydrochloride (DAPI, Sigma) was applied to stain the cell nuclei. Finally, stained cells were imaged using a confocal microscope (Leica SP8X DLS, Germany), and images were analyzed using the ImageJ software (the USA).

### 2.4 Fabrication and characterization of PCL-GelMA hydrogel hybrid scaffolds and skin model

#### 2.4.1 Preparation of PCL/GelMA composite

PCL scaffolds with three dermal designs (192 layers for convenience in the design software) and an epidermal layer (20 layers) were printed. To enhance the hydrophilicity of PCL scaffolds, they were etched with 5 mol/L NaOH for 2 h, followed by washing 10 times (30 min each time) with deionized water. Water contact angle (DataPhysics OCA30, the USA), and FTIR (Bruker IFS88, USA) were utilized to compare the wettability and molecular structure of untreated and etched PCL scaffolds. The results are provided in Figure SI 3. Next, based on the preliminary findings from compressive tests and cell studies, the etched PCL scaffolds were immersed into 5 % (w/v) GelMA solution (100 μL: 120 mm^3^) containing the photoinitiator LAP (0.25 % (w/v)) at 37 °C, and subsequently crosslinked using 405-nm UV light for 10s. Before immersion, GelMA was stained with Tiffany Blue dye to visualize its location within the PCL scaffolds (Fig. SI 5). ESEM images of the PCL/GelMA composite were captured from both the top and bottom sides at 3 °C and 10 kV accelerating voltage to visualize the dispersion of GelMA during dehydration of the composite in situ. Therefore, the water vapor pressure inside the SEM chamber was changed from 750 Pa to 350 Pa (corresponding to a change from 100 % to 47 % relative humidity at 3 °C).

Tensile tests of the pure PCL scaffolds with three dermal designs and the corresponding PCL/GelMA composites at day 1 were conducted using a tensile tester (Instron 5565 100N Series IX, the US) with a 0.5 kN load cell at an elongation speed of 10 mm/min. Three samples were measured per group to collect the average Young's modulus. Corresponding compressive measurements were performed using the same LLCT instrument and parameters mentioned in **2.3.2.1**.

#### 2.4.2 Degradation tests of PCL/GelMA composites

Degradation experiments were performed to assess the stability of crosslinked PCL/GelMA composites. UV irradiation was performed at a height of 10 cm above the samples, the crosslinking time was 10s. Samples were placed in the 48-well plates, 1 mL DPBS was added to each sample, and the plate was placed at 37 °C in an incubator. The degradation was analyzed at three different time points: 0 days, 7 days, and 14 days. At each time point, a separate set of samples was washed using deionized water and dried in the oven overnight. The mass of each sample was collected. The average weight loss was calculated and recorded.

#### 2.4.3 SE preparation and RTA treatment on SEs

Human epidermal keratinocytes (HaCaTs) were purchased from CLS Cell Lines Service (CLS, the USA), and maintained in HEPES buffered Dulbecco's modified Eagle medium (DMEM, high glucose, GlutaMAX™ Supplement, Gibco, the USA) supplemented by 10 % fetal bovine serum (FBS, Gibco, the USA), 1v/v % Penicillin-Streptomycin and 1v/v% Amphotericin B. Human adult dermal fibroblasts (NHDF-Ad; Lonza, The Netherlands) were cultured in the same medium. All cell flasks and well plates were placed in a humidified incubator at 37 °C, 5 % CO_2_.

After harvesting, NHDF-Ad cells were counted using a cytometer and centrifugated at 1000 RPM for 5 min. Cells were resuspended in sterilized GelMA solution at a density of 1 x 10^7^ cells/mL. The initial etched PCL scaffolds (20 mm × 20 mm x 1.6 mm) were cut into 1.1 mm^2^ pieces followed by sterilization with 70 % ethanol for 15 min in 48-well plates. Dried PCL scaffolds after etching were immersed in GelMA suspension (150 μL for each sample) containing cells. These composite scaffolds were transferred to new well-plates and crosslinked under 10s UV irradiation. Next, 200 μL HaCaT suspension in medium (10^8^ cells/mL) was loaded on the epidermal layer of the PCL/GelMA composite. The samples in 48-well plates were gently put into an incubator and kept overnight. During this time the HaCaTs could attach to the scaffold fibers. Next, 2 mL medium with 5 % FBS was carefully added into each well for the following co-culture. After 3 days, the samples were transferred to 12-well plates, and the top interface (keratinocyte layer) of the hybrid composites was lifted into the air by adding the medium to the height just below the composites. This allowed the keratinocyte layer to be exposed to air and induce the cornification of keratinocytes. Cells were cultured in the lifted samples for the next 4 days.

The selected SEs (3 samples) based on the previous cellular behavior were treated with 100 μM RTA during the airlifting process. Specifically, RTA was first dissolved in DMSO (15 mg/mL, Sigma) and filtered with 0.45-μm sterile filters. Then it was diluted into the cell medium to a final concentration of 100 μM. The RTA-containing medium was added during the 10-day coculture.

Compressive measurements of the SEs after 7-day coculture with and without RTA treatment were performed using the same LLCT instrument, and the parameters were mentioned in **2.3.2.1**. Three samples were measured per group to collect the average moduli.

#### 2.4.4 Human skin samples

For comparison with the artificial skin model, we used human skin samples from patients who underwent plastic surgery at the insert department, UMCG. Ethical considerations were cautiously addressed. Approval from the Institutional Medical Ethical Review Board was sought and subsequently waived (Reference No. M24.332256), as the study employed anonymized waste material. Necessary guidelines were followed to use these samples in the main manuscript. More specifically, we used the waste material consisting of skin tissue from the patient's stomach which was removed during surgery within the standard clinical care. All participating patients provided informed consent, with their materials processed anonymously.

##### 2.4.4.1 Cryosection

For histological investigation, the skin grafts and human skin samples as control were embedded in an Optimal Cutting Temperature compound (O.C.T., Fisher Scientific, the USA) and then frozen with liquid nitrogen. The samples were sliced into 10-μm thick sections using the cryostat (Leica CM 1950, Germany) following the cryosection procedure.

##### 2.4.4.2 H&E staining, immunohistology, image analysis and enzyme-linked immunosorbent assay (ELISA) tests

The slices were treated with acetone for 10 min to remove the O.C.T. compound and to fix the samples. Then, the sample slides were stained with hematoxylin for 5 min and washed with tap water for 5 min. Slices were then transferred to Scott's blue for another 5 min, dipped in tap water, and incubated for 20 s with eosin staining solution. After staining, the slices were observed under an optical microscope.

For immunohistological analysis, the acetone-treated slices were rinsed with DPBS three times and treated with permeabilization solution (Triton 100x, 0.05 %) for 10 min. After washing three times with DPBS, the slices were incubated with 5 % BSA to block non-specific sites for 1 h. Skin sections were incubated with Anti-keratin 14, 10 antibodies (1:500 in PBS, Abcam), Anti-Collagen IV and I antibody (1:200 in DPBS, Fisher Scientific), and Anti-Vimentin antibody (1:200, Sigma), Anti-Elastin (1:40, Novotec), Anti-Fibrillin (1:200, Millipore) at 4 °C overnight respectively. After washing three times (5 min each time), secondary antibodies (Alexa Fluor® 561, 647 respectively) were added for 45 min at RT. Nuclei were stained with DAPI and skin section samples were imaged using a Zeiss confocal microscope (Leica SP8X DLS, Germany). Images were analyzed using ImageJ (the USA).

After a 6-day culture, the medium of the SEs, both with and without RTA treatment, was replaced with fresh medium. One day later, the supernatants from the samples were collected, centrifuged, and stored at −20 °C for subsequent analysis. Protein levels of human procollagen Iα1, elastin, and fibrillin were quantified using the respective ELISA kits (R&D Systems, Abcam, and antibodies respectively, the Netherlands) according to the manufacturer's instructions. Absorbance readings were obtained using a microplate fluorometer (Thermo Scientific Fluoroskan, Netherlands). Total protein concentrations were calculated following the manufacturer's protocol.

### 2.5 Integration of cellular tubular structure

The PCL tubular structure at a small diameter (ca. 1 mm) was printed by the same MEW instrument as mentioned in **2.1.** On the collector plate, a custom-built mandrel setup was placed. The mandrel consisting of a glass rod with a diameter of 1 mm attached to NEMA 17 motor, was controlled using an Arduino UNO board (Italy). A custom code was used to operate the rotational speed of the mandrel. The translational speed was set to 60 mm/min, and the printing pressure and voltage were set at 0.5 bar and 13.5 kV, the rotational velocity of the rod was set at 600 rpm. The final dimensions of the printed tubular structure were ca. 20 mm x Φ1.1 mm with 10 printed layers.

The obtained tubular scaffolds were etched following the protocol in Section 2.4.1. Then, after a 15-min sterilization in 70 % ethanol, the tubular structures were immersed in a 1 % gelatin solution for 30 min and then dried. Human Umbilical Vein Endothelial Cells (HUVECs) provided by the Endothelial Cell (EC) Facility at UMCG were used for seeding. The cells were cultured in EC medium composed of RPMI 1640 Medium containing 20 % heat-inactivated FBS, 50 μg/mL Gentamycin, 2.9 mg/mL L-Glutamine, 5 U/mL Heparin, and 30 μg/mL EC growth factors. After 3 days of culture, the cells were harvested and dissolved in Matrigel at a concentration of 1 x 10^7^ cells/mL. The tubular scaffolds were immersed in a HUVEC-containing Matrigel (20 v/v % Matrigel) suspension for 15 min. Next, the cellular composites were incubated for 10 min to allow thermal crosslinking, followed by the addition of the EC medium. The composites were then cultured for 3 days. Mechanical properties, including the tensile modulus and compressive modulus of the cellular tubular grafts after 3 days of culture, were tested following the procedures described in Method Sections 2.3.2.1, 2.4.1.

For cellular tubular structures incorporation, the SEs scaffolds were prepared 6 days in advance following section 2.4.3 with an alternation in the scaffold printing procedure Specifically, the dermal layer was fabricated with the insertion of the 2-mm rod, providing the channel for the later incorporation of the cellular tubular structure. In short, after printing half of the dermal layer, the process was stopped, the 2-mm glass rod was deposited on top of the structure and the remaining fibers were printed on top of the rod. After 6-days cell culture, the rod was removed. Then with the support of a 1 mm-diameter needle inside the tubular graft, the cellular tubular structure was integrated into the SE scaffold, without adhering to the channel walls, and the empty space between scaffolds and tubular graft was filled with 50 v/v % Matrigel. The construct was cocultured in EC medium for 1 day. Before switching from the normal medium to the EC medium for culturing SEs with vascular structures, we tested the metabolism and spreading behavior of fibroblasts and keratinocytes in the EC medium over a 7-day period, using cells in the normal medium as a control group. No significant difference in metabolism was observed (Fig. SI 9a), and the cells spread well in both media (Fig. SI 9a). Thus, the cell functions of the skin cells were not compromised by using the EC medium for 1-day co-culture. The efficacy of RTA at 100 μM on vascular structure development within the SEs was studied during the 4-day culture.

The simplified perfusion test for the vascular grafts within the SEs was conducted following the protocols outlined in previous studies [[70], [71], [72]]. Shortly, the cell medium was pumped through the vascular grafts, flowing from one side of the SEs to the other (see the video in SI information).

The morphology and crossection of the tubular structure, and tubular structure within SE scaffolds were analyzed by the SEM as mentioned in **2.1**. Cryosectioning and histological staining of SEs containing tubular grafts before and after RTA treatment were carried out using the previously mentioned protocol (see section 2.4.4.1, 2.4.4.2). The CD31 biomarker (Abcam) was used to stain the HUVEC junctions within the cellular tubular structure. After staining, the samples were analyzed using optical or confocal imaging, with subsequent analysis conducted using ImageJ.

### 2.6 Statistical analysis

The results are reported as mean and standard deviation. One-way variance (ANOVA) and Tukey's honestly significant difference (HSD) post-hoc test were used to analyze the significance between the means of different samples. Data analysis was performed using GraphPad Prism software (GraphPad Software Inc., the USA). ∗ indicates p < 0.05, ∗∗ means p < 0.01, ∗∗∗ means p < 0.001 and ∗∗∗∗ means p < 0.0001.

## 3 Results and discussion

### 3.1 PCL scaffolds

Skin tissue is a complex structure comprising multiple physicochemical gradients to fulfill various functional requirements [73]. Thus, multilayer scaffolds have gained great attention in mimicking the real skin environment [74,75]. In this study, to closely mimic the skin tissue, structures composed of two zones: epidermal and dermal layers were printed (see Fig. 1, Fig. 2b, c, and d). Three designs of dermal layers were proposed (Fig. 2 b, c, and d: dermal layer), composed of 192 layers (192 layers for convenience in the design software) of fibers resulting in the same total thickness (ca. 1.25 mm), but different pore sizes and shapes, namely Cage 1, Cage 2, and Triangle (the parameters of porosity, fibre size, and density are collected in Fig. 2e). The Cage 1 structure, with pore sizes ranging from 0.012 to 0.28 mm^2^, has the highest porosity (92 %). The big pore sizes and high porosity may benefit the incorporation and dispersion of fibroblast -laden gel due to relatively fewer fibers, thereby enhancing the homogeneity of cell distribution. According to a previous study, the preferred porosity of scaffolds for cell penetration is above 60 % [76]. The high porosity of Cage 1 may facilitate cell penetration. The density of Cage 1 is 0.06 g/cm. The Cage 2 structure has the same fiber orientation and design as Cage 1 but features smaller pore sizes ranging from 0.01 to 0.067 mm^2^. With a slightly lower porosity of 89.1 %, the scaffold density of 0.095 g/cm^3^ potentially hinders cell penetration, dispersion, and migration compared to Cage 1. The triangle structure features much smaller pore sizes (0.0009–0.004 mm^2^), with a porosity of 84.2 %. The scaffold density (0.22 g/cm^3^) is much higher than that of Cage 1 and 2, indicating a greater likelihood of limiting cell penetration and migration between the epidermal and dermal layers, as well as within the dermal compartment. Additionally, the smaller pore size and lower porosity may significantly limit cell dispersion. The fibre sizes of the 3 structures are similar (ca. 16 μm) as measured from the SEM images. After printing dermis layers, we constructed the same epidermis layer on top of the 3 dermal designs. The epidermal layer was composed of random fibres with sizes ranging from ca. 4 μm to ca. 150 μm, with the total layer thickness of ca. 0.35 mm. This layer was used to support the homogeneous seeding of keratinocytes (shown in Fig. 1c), their orientation, and to hinder their migration into the dermis layer [77].

After printing, the constructs were treated with 5M NaOH to increase the hydrophilicity. FTIR was performed to analyze the molecular structure of untreated PCL and alkali-etched PCL scaffolds. The FTIR graph (Fig. SI 4a) reveals almost no difference in the characteristic peaks between the etched and nontreated PCL scaffolds [78], which may be attributed to the relatively small amount of modified material on the fiber surface when compared to bulk non-modified material inside the fibres. To check if alkali etching increased the hydrophilicity of the scaffolds, we performed water contact angle measurements. After etching, the water contact angles were reduced from ca. 115° to below 60° (Cage 1: 44.7°, Cage 2: 49.2°, Triangle: 53.2°). This indicates the high surface hydrophilicity (Fig. SI 4b) [79]. The etching method for PCL using NaOH was reported before [80,81]. The improved hydrophilicity could benefit the hydrogel incorporation and cell seeding [82].

### 3.2 Characterization of hydrogels

Before casting the fibroblast-laden gels into PCL scaffolds, the characterization of hydrogels was conducted.

#### 3.2.1 FTIR and 1H NMR analysis

FTIR measurements were carried out to confirm the methacrylation and analyze the chemical structure of the GelMA. The FTIR (Fig. 3b) shows characteristic peaks in the structure of pure gelatin and functionalized gelatin. The specific vibration peaks of functionalized gelatin are present in the spectra at 3330 cm^−1^, 3076 cm^−1^, 2943 cm^−1^ and 1679 cm^−1^ which are assigned to the signals for O–H and N–H stretching, saturated C–H stretching, and amide I, respectively [83,84], changes in distinguished peaks indicate the successful methacrylation of gelatine. The methacrylation in GelMA was further quantiﬁed using 1H NMR spectroscopy. The peaks between 5 and 6 ppm on the spectra were due to acrylic protons from the methacrylate, and the peaks between 1.5 and 2 ppm were due to the methyl function from the methacrylate [85]. As can be seen in Fig. 3c, the amount of amino lysine in GelMA (2.8–2.95 ppm) was significantly decreased, indicating the obvious methacrylation. We further quantified the 1H NMR data and found that the degree of methacrylation was 24 %.

#### 3.2.2 Compressive moduli of GelMA under different UV exposure times and Matrigel at 50, 75, and 100 v/v %

It is reported that the elastic modulus of GelMA can be modulated by changes in the UV irradiation time [86]. Therefore, we tested the stiffness of 5 w/v.% GelMA after different crosslinking times, namely, 10 s, 15 s, 20 s, 30 s, 40 s and 60 s UV curing time. To compare with Matrigel, different concentrations of Matrigel (50, 75, and 100 v/v %) were also measured. The maximum compression of Matrigel (100 v/v %) is 0.912 kPa, reaching a similar modulus of 10 s crosslinked GelMA (0.887 kPa). From 10 to 40 s, there is a significant increase in compressive moduli (Fig. 3d). The same moduli of the 40 s and 60 s GelMA indicate that most of the bonds are already formed, and the effect of UV light exposure does not further increase after longer irradiation times (over 40 s).

#### 3.2.3 Cell viability, proliferation, spreading and ECM deposition behavior in hydrogel

Cell viability in 5 % GelMA was tested to analyze GelMA hydrogel's biocompatibility after 10 s, 20 s, and 40 s of UV treatment. NHDF cell viability at ca. 88 % was observed for all tested groups, indicating that exposure times up to 40 s UV curing will not compromise the cell survival rates (Fig. SI 1).

To assess cellular behaviour in two hydrogels with similar compressive moduli, cell performance in GelMA crosslinked for 10 s and undiluted (v/v) Matrigel was compared. Cell performance in GelMA crosslinked for 20 s and 40 s was further investigated to evaluate how variations in stiffness and UV irradiation time affect cell behavior in GelMA. Cell metabolism in the above-mentioned Matrigel and GelMA were compared within one week of cultivation using the AlarmaBlue kit. A significant increase in the metabolism of GelMA after 10-s crosslinking (GelMA-10s) was found when compared with all other experimental groups within 7-day culture (Fig. 3f), indicating the GelMA-10s was the most promising condition.

The cell shape and expression of migration protein (Vimentin), ECM remodeling protein (Collagen I), and morphology protein (F-actin) were compared through immunostaining at day 7 and 14 cultures (Fig. SI 2 and 3). Vimentin is the intermediate filament protein identified in NHDF cells that plays a crucial structural role in maintaining cell shape and integrity [87,88]. It is required for many vital cell functions like cell motility, chemotactic migration, and wound healing [89]. As visible in Fig. SI 2, Vimentin was prominently expressed in the GelMA after the 10-s crosslinking group compared to other groups on day 7. Additionally, Collagen I, which is a crucial component in skin remodeling, showed a more homogeneous and denser expression and organization in the GelMA-10s group. The F-actin staining revealed well-distributed spindle-shaped cell morphology and a larger spreading area in the GelMA-10s group (Fig. SI 2) when compared to GelMA after 20-s, 40-s crosslinking (GelMA-20s, GelMA-40s), and Matrigel (100 v/v %). The orientation and shape of cell nuclei (elliptical) aligned with the cell spreading direction, and the number of cells indicated by the cell nuclei in this group was higher than in other groups (Fig. SI 2), confirming higher cell proliferation (Fig. 3f). Representative images of the GelMA-10s group are shown in Fig. 3h, with cells on Petri dishes as controls (Fig. 3g). After 2 weeks of culture in the gels, no significant differences were observed among all tested GelMA groups (Fig. SI 3). This result aligns with previous studies, which showed that cells encapsulated in GelMA with lower stiffness crosslinked in a shorter time (10 s) could quickly start spreading to form interconnected cellular networks [69]. We observed that the growth of cells in Matrigel was slower than in GelMA within 2 weeks (Fig. SI 2 and 3, Fig. 3f). This is possibly due to the high concentration of Matrigel resulting in lower porosity and limited nutrient transfer. Therefore, the GelMA-10s group was selected for subsequent skin regeneration research due to its optimal cell behavior.

### 3.3 Fabrication and characterization of composite scaffolds

**Fig. 4:**
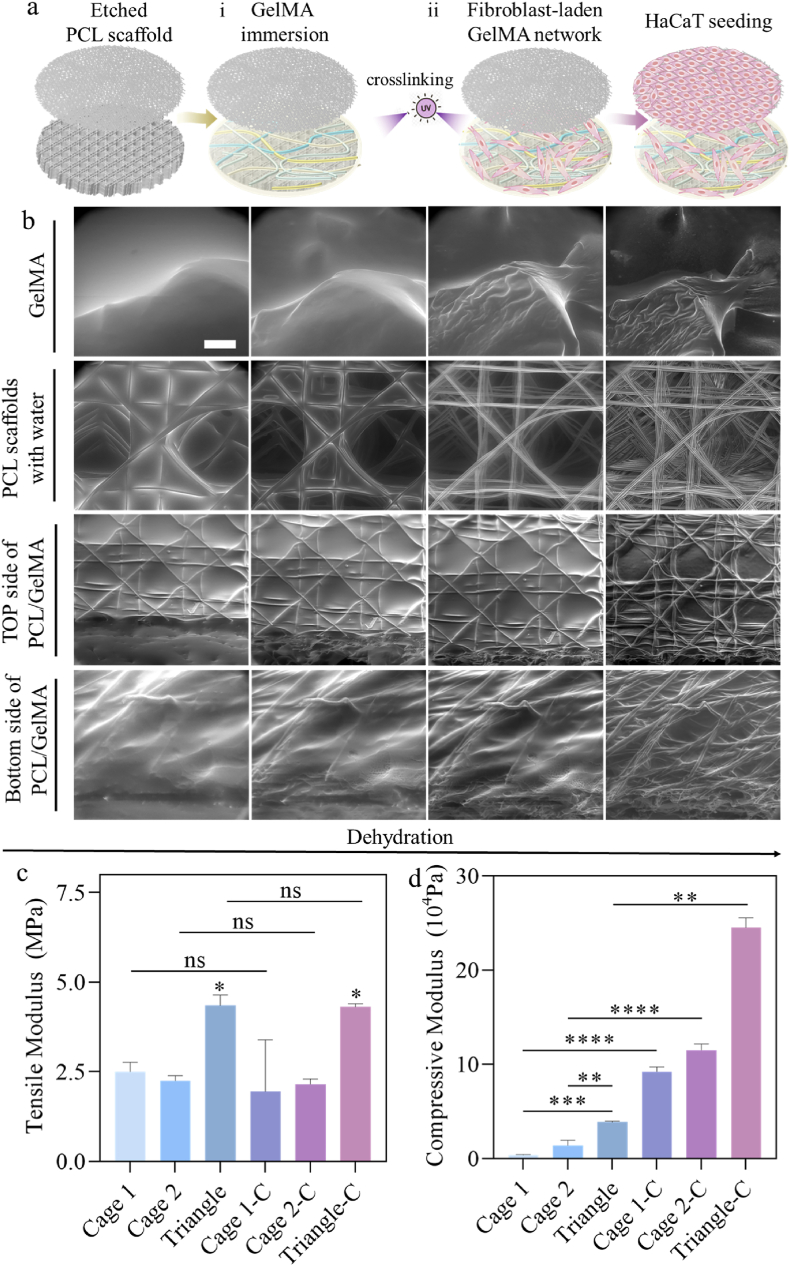
PCL/GelMA composites characterization. a) Schematics showing fabrication of i) PCL/GelMA composites by immersing the etched PCL scaffolds in GelMA solution, ii) bilayer cellular PCL/GelMA composites. b) In-situ ESEM images of the acellular composites changing state from wet to dry in time (750 Pa, 600 Pa, 450 Pa, 350 Pa, respectively). Full integration of the PCL scaffold (Cage 2 as an example) and GelMA hydrogel is visible. Scale bar: 100 μm. c) Tensile, and d) compressive moduli observed for Cage 1, 2, and Triangle PCL scaffolds and corresponding PCL/GelMA composites (named Cage 1-C, Cage 2-C, and Triangle-C respectively) at day 1. The data are shown as Mean ± SD. p value ∗≤ 0.05, ∗∗≤ 0.005, ∗∗∗≤ 0.001, and ∗∗∗∗≤ 0.0001, ns: p ≥ 0.05, one-way ANOVA tests, n: 3.

The PCL/GelMA composites were prepared by immersing PCL scaffolds in GelMA

solution mixed with a Tiffany blue coloring agent, followed by 10 s of UV crosslinking (Fig. 4a). Optical images of stained PCL/GelMA composites (Fig. SI 5) show the homogenous distribution of GelMA (stained in blue) within PCL scaffolds. To further investigate the gel penetration and distribution in the PCL scaffolds, in situ SEM images were collected from the wet to dry state of the composite (Fig. 4b). Fig. 4b further confirmed that GelMA was well distributed in the scaffold. There was no uncovered space, and, importantly, there was no deformation observed in the PCL scaffold after the combination with GelMA. These observations of the penetration of hydrogel into PCL scaffolds and the successful material combination are corroborated by previous work by Castilho et al. [49] Besides, we hypothesized that MEW mesh reinforcement can prevent gel shrinkage in the cell culture.

The degradation measurements were performed to test the stability of PCL/GelMA within 2 weeks. The weight loss is summarized in SI Table 1. The data indicates that slight degradation (<2 wt%) was observed within 2 weeks of incubation at 37 °C, showing the high stability of the PCL/GelMA composites during the incubation process.

To obtain mechanical properties matching native skin, GelMA was combined into multi-layered PCL scaffolds. The composites were prepared by immersing the dermal printed layers in GelMA solution, followed by UV crosslinking for 10 s. Mechanical evaluation including tensile and compressive moduli of the composite constructs was further performed to compare the materials. The tensile moduli of PCL scaffolds and PCL/GelMA composites on day 1 (Fig. 4c) did not present significant differences. We hypothesize that the tensile modulus of the composite purely depends on the reinforcing structure of the MEW mesh [90]. Mouser et al. also reported no increase in tensile Young's moduli when PCL scaffolds were infused with methacrylated hyaluronic acid [91]. The measured tensile moduli of Cage 1 and 2 are ca. 2 MPa (Fig. 4c), which is close to that of the human skin (0.5−1.95 MPa) [92]. Whereas the tensile moduli of triangle designs are over 4 MPa, which can be attributed to the denser fibres.

The compressive moduli of the experimental groups are summarized in Fig. 4d. Interestingly, the compression modulus of Cage 1 increased from 3.8 kPa (pure PCL scaffolds) to 92.1 kPa, achieving a maximum reinforcement of 24.2-fold. Cage 2, with an initial stiffness of 13.8 kPa due to its higher scaffold density, exhibited a compressive modulus of approximately 115 kPa after embedding GelMA, indicating an 8.3-fold increase. The triangle structure, with the highest density and an initial compressive modulus of approximately 38.5 kPa, reached a final modulus of around 245 kPa after incorporating GelMA. This increase caused by the introduction of GelMA aligns with reports by Visser et al. [42] and Castilho et al. [43], who showed that the compression modulus can increase by up to 54-fold and 47-fold respectively, when GelMA is incorporated into PCL scaffolds without cells. This improvement is primarily attributed to the prevention of structural buckling. The obtained compressive moduli fall within the range typical for human skin, which is between 60 and 400 kPa [92]. These observations further confirm the suitability of our composite for potential applications in skin tissue engineering and regeneration.

### 3.4 Analysis of cell penetration, distribution, and infiltration in the cellular PCL/GelMA composites

**Fig. 5:**
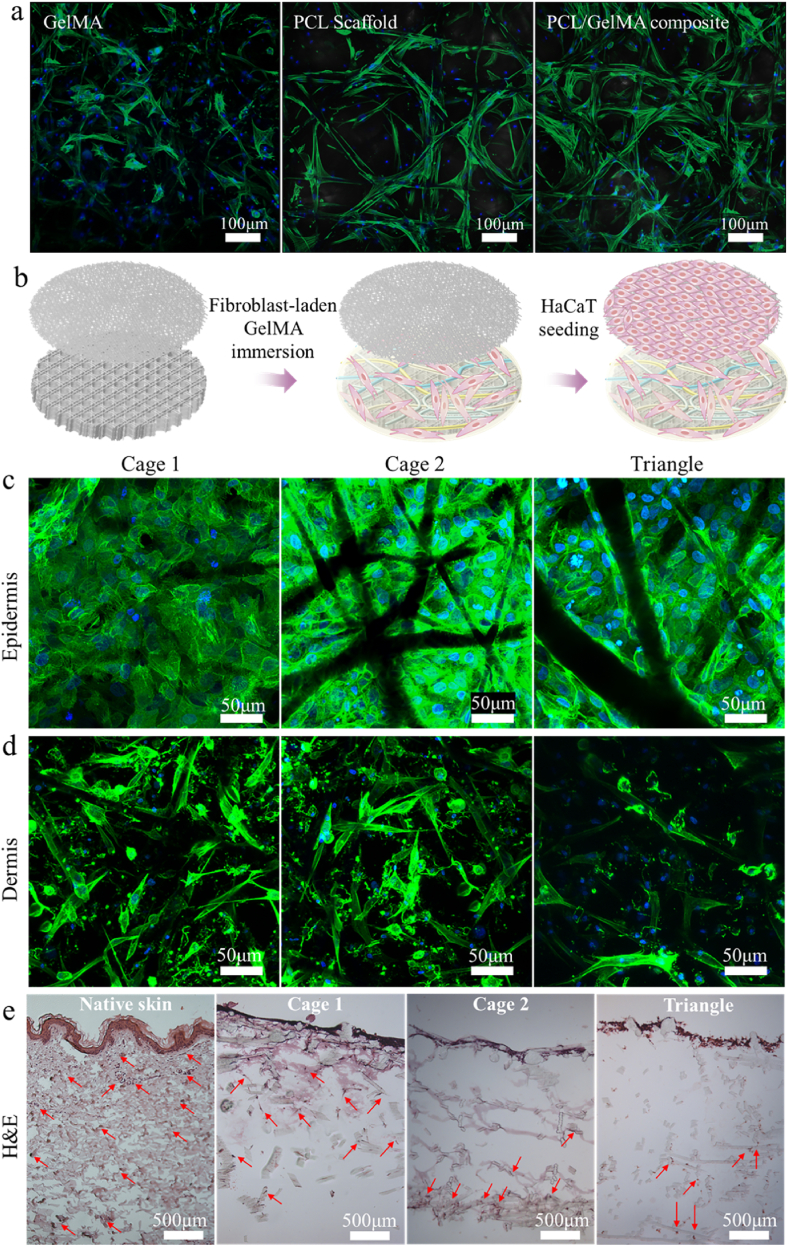
a) Comparison of cell performance in different scaffolds: Z-stack images of cell spreading and distribution after 1-week culture in: pure GelMA, PCL scaffold, PCL scaffolds immersed in GelMA b) Schematics of the fabrication of bilayer cellular PCL/GelMA composites. The cell morphology and distribution of c) HaCaT in the epidermal layers and d) NHDF at the bottom side of the dermal compartment in the scaffolds. F-actin was labelled in green and nuclei were stained in blue. e) H&E staining of the human skin (control) and the section slices of Cage 1, Cage 2, and Triangle composite designs. Cell nuclei are purplish blue, cytoplasmic components are pink. 3 samples were imaged. Scale bars: a) 100, c) 50, d) 50, and e) 500 μm. (For interpretation of the references to color in this figure legend, the reader is referred to the Web version of this article.)

To compare cells spreading in GelMA, PCL scaffolds, and PCL/GelMA composites, the cell cytoskeleton was stained with Phalloidin-FITC and DAPI (Fig. 5a). Cells within the GelMA group demonstrated a relatively anisotropic spreading when compared to the other two groups (cells in PCL scaffolds, PCL/GelMA composites). Within PCL scaffolds and PCL/GelMA composites, cells tended to elongate along the PCL fibers or grow across the pores. Interestingly, in PCL/GelMA composites, in comparison with the pure PCL scaffold, there were more cells and they spread homogeneously in the pores and on the scaffold after a 7-day culture. We concluded that the composite's environment facilitates the formation of a typical spindle shape of the cell, aided by the presence of fibers. The results suggest that conducting 3D cell culture within the composite not only keeps the cells in the construct but also closely mimics the cell shape found in real tissue [93].

Further, to compare the influence of scaffold designs on cell penetration, distribution, and migration in the cellular composites, which consisted of a fibroblast-laden dermal layer and a HaCaT-seeded epidermal layer (Fig. 5b), and the cell cytoskeleton was stained. Cryosections of the composites were stained with H&E agents. Epidermis and dermis were well-formed after 1-week co-culture based on the observation from the fluorescent and H&E staining images (Fig. 5c and d). Well-spread keratinocytes were stacked on the epidermal layer (the dark area is PCL fibers), whose thickness is ca. 150 μm; and spindle-shaped fibroblasts were observed at the bottom of the dermal compartment at the thickness of around 1 mm. To assess cell penetration, distribution, and migration within the dermal layers and between the epidermal and dermal layers, H&E staining images of the slices were collected. Human skin donated at the University Medical Center Groningen was used as a control group for comparison. Compared to native skin, all artificial skin composites exhibit a comparable architecture after 1-week co-culture. In the epidermis, several layers of living cells can be identified in both the skin models and the human skin. The interface between the epidermis and dermis was clear, indicating no significant cell infiltration between these two layers. In the dermis, fibroblasts in Cage 1 were distributed just below the epidermis and penetrated throughout the dermal layer (labelled by red arrows, Fig. 5e, Cage 1). However, in structures with smaller pore sizes and lower porosities, such as Fig. 5e, Cage 2 and Triangle, fibroblasts were observed stacked or distributed near the bottom (marked with red arrows), suggesting that inherently smaller pores did not promote adequate cellular infiltration, penetration, and dispersion in the scaffolds, which is particularly essential in skin engineering [94,95]. Based on the obtained results, Cage 1 was selected to fabricate and functionalize the skin replicates in the following study.

### 3.5 H&E staining and immunostaining of the skin equivalents

**Fig. 6:**
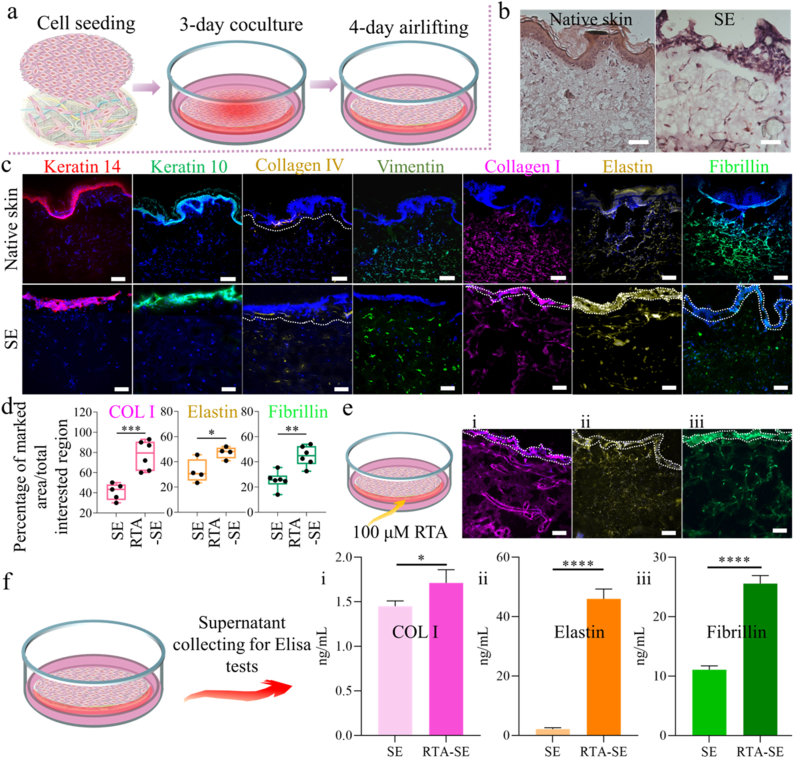
a) Schematics of the fabrication process of SE. b) H&E staining of the human skin (control) and the section slices of Cage 1-SE. Cell nuclei are purplish blue, cytoplasmic components are pink. c) Biomarker analysis of native human skin samples and developed skin models. Cell nuclei were stained with DAPI (blue), and basal and stratum spinosum layers were labelled by Keratin 14 (red), and Keratin 10 (green). Cell junction components were labelled by Collagen IV (yellow). The dermis was labelled by Collagen I (magenta), vimentin (green), and Elastin (yellow), Fibrillin (green). d) Immunostaining image analysis after RTA treatment: percentage of the total region of interest occupied by Collagen I, Elastin, and Fibrillin. e) (i) schematics of the experiments and the expression of Collagen I, (ii) Elastin, and (iii) Fibrillin after RTA treatment. Epidermal layers in c) and e) were highlighted by white random circles. Significant variation was not observed in the epidermis thickness. f) Collagen I, Elastin, and Fibrillin quantified by ELISA after RTA treatment. p value ∗≤ 0.05, ∗∗≤ 0.005, ∗∗∗≤ 0.001, and ∗∗∗∗≤ 0.0001, t tests, n: 3–6. Scale bars: 100 μm. (For interpretation of the references to color in this figure legend, the reader is referred to the Web version of this article.)

To confirm the formation of a SE, after airlifting shown in Fig. 6a, the skin slices were analyzed by H&E and immunostaining. The H&E staining images confirmed that the epidermis and the dermis were well-formed after 7-day of co-culture (Fig. 6b), and the thickness of the epidermis and dermis in SE samples was similar to native skin. The interface between the epidermis and dermis was very clear. The epidermis was organized into a three-dimensional lattice of tightly adhering cells, and the waved architecture of the epidermis closely mimicked that of native skin, distinguishing it from previous flat artificial epidermis designs [1,34,52,96]. The cell density of HaCaTs in the obtained SEs was similar to the human skin (namely, 27 ± 3 cells/5000 μm^2^, 30 ± 4 cells/5000 μm^2^, respectively). The cellular architecture of the epidermis is essential for its barrier and protective functions [97]. The sections of the PCL fibers are highlighted with white arrows (Fig. 6b). The fibroblasts formed spindle shapes in the artificial dermis (marked with black arrows in Fig. 6b-‘SE’), which is similar to the real dermis. Compared to the native skin dermis, in our artificial skin, the fibroblast cell densities were also similar (namely, 15 ± 3 cells/10^7^ μm^2^ in SE versus 16 ± 2 cells/10^7^ μm^2^ native skin. The histological analysis confirms the successful skin formation utilizing the composite scaffolds.

To further characterize the obtained SEs (Cage 1 design), the slices of the SEs were stained for the expression of various biomarkers: Cytokeratin 14 and Cytokeratin 10 for HaCaTs (keratinocytes), Collagen IV for the epidermal-dermal junction, and Collagen I, Vimentin, Elastin, and Fibrillin for NHDF (fibroblasts). Additionally, nuclei were stained to locate the cells, as shown in Fig. 6. The epidermal compartment showed expression of Keratin 14 and Keratin 10 in the keratinocyte layers, corresponding to the basal layer and stratum spinosum, respectively (Fig. 6c). This revealed a properly stratified epidermis with a well-organized basal and stratum spinosum layer. The dermal-epidermal junction, a distinctive feature of skin tissue, is characterized by the presence of Collagen IV [37,96]. The positive Collagen IV expression in both control and artificial skin indicated the well-connected epidermal and dermal layers. Immunostaining images of Vimentin revealed a uniform expansion and alignment of spindle-shaped fibroblasts within the dermal compartment. Some fibroblast cells were situated near the basal membrane region beneath the epidermis. This is similar to the human skin (shown in Fig. 6c). The staining of Collagen I revealed the homogeneously organized dermal matrix. The well-arranged Collagen I indicated that the proposed design of the dermal printed structure is suitable for ECM neosynthesis and organization. Furthermore, Elastin and Fibrillin were observed, and the immunofluorescence of these two biomarkers also revealed the homogenously distributed fibroblasts and well-organized ECM (Fig. 6b). Overall, the labeling by biomarkers in the epidermis, conjugation layer, and dermal layer exhibited a close resemblance to native skin. RTA facilitates epidermal cell differentiation [98], resulting in increased epidermal thickness and modulation of epidermal morphogenesis [99,100]. Additionally, RTA promotes ECM production by stimulating the synthesis of key ECM components, including Collagen I, Elastin, and Fibrillin [[101], [102], [103]]. Furthermore, RTA can inhibit endothelial cell growth and proliferation, which may contribute to the maturation and stabilization of fully formed, quiescent blood vessels.

[104,105] Based on these previous findings, the influence of RTA on the epidermis, dermis within the SEs, and vascular structure within the SEs was investigated. The above-mentioned samples were treated with 100 μM RTA during the culture process; this concentration was optimized in the previous study by Pourjafar et al. [106] From the fluorescent images (Fig. 6c and e, epidermis marked with white dashed circles), no significant difference in epidermal thickness was observed before and after RTA treatment. This outcome may be attributed to the high initial cell number (2 x 10^7^ cells/mL), which likely facilitated the rapid formation of the epidermal layer. As a result, the additional thickness resulting from cell proliferation and cornification may have been minimal or negligible. In the dermis, the immunostaining images of Collagen I, Elastin, and Fibrillin confirmed increased expression after RTA treatment, and the occupancy of the biomarker expression significantly increased from 41.2 %, 32.5 %, and 25.4 %–77.3 %, 47.34 %, and 44.8 % respectively (Fig. 6 d-COL I, Elastin, Fibrillin, e-i, ii, iii). The quantification of Collagen I, Elastin, and Fibrillin further demonstrate the enhanced expression following RTA treatment. The compression moduli of SEs and RTA-SEs show no significant difference, both around 100 kPa, though RTA-SEs exhibit a slight increase in compressive moduli (Fig. SI 8). This result aligns with previous studies that highlight RTA's role in facilitating ECM reconstruction [101,107,108], further proving that SEs can serve as an effective platform for testing.

**Fig. 7:**
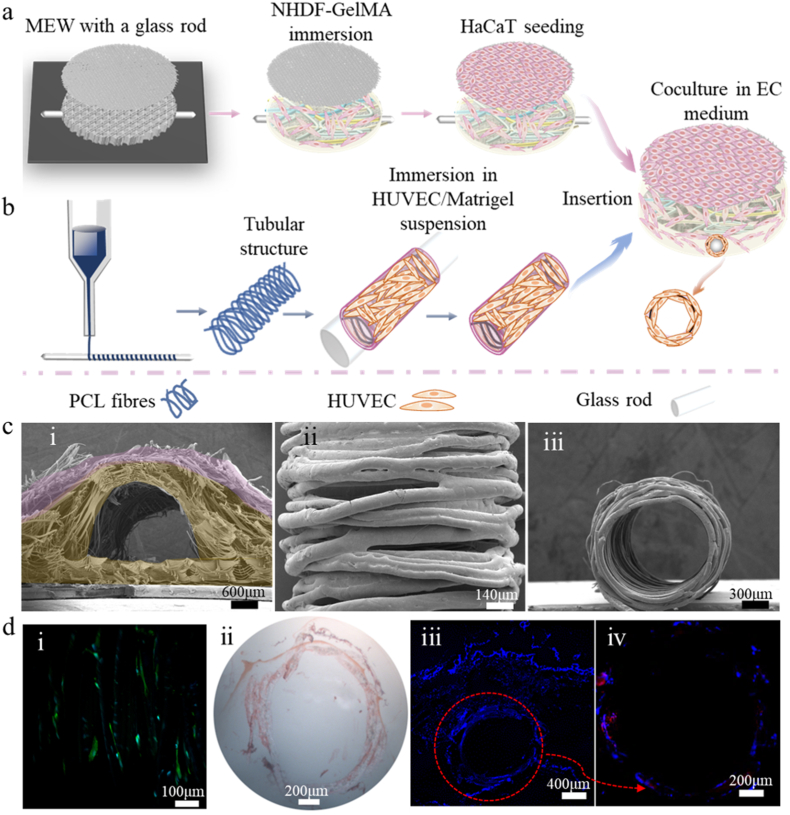
a, b) Schematics of the fabrication process of SEs with cellular tubular structure. a) Scaffold printing with the help of a 2 mm glass rod, and the preparation of SEs with the rod. b) Printing of tubular structure and the combination of HUVEC-laden Matrigel layer. c) SEM images of the i) SE scaffold with a channel; ii) the top view of the tubular structure; and iii) the cross-section of the tubular structure. d) Cellular behavior on tubular structure: i) cytoskeleton staining of HUVEC, nuclei are in blue, F-actin is in green; ii) H&E staining of the slices, nuclei are purplish blue, cytoplasmic components are pink; iii) nuclei distribution in SEs, the cellular “ring” is visible; iv) expression of CD31 (Red) in HUVEC “ring”. 3 samples were analyzed. Scale bars are shown above. (For interpretation of the references to color in this figure legend, the reader is referred to the Web version of this article.)

Additionally, a tubular structure with an outer layer of HUVECs embedded in Matrigel was prepared to mimic the blood vessel. A channel of approximately 2 mm in diameter (Φ 2 mm) was left in the printed SE scaffolds to integrate the cellular tubular structure (Fig. 7a, b present the fabrication process). The resulting SE scaffold with the channel is shown in Fig. 7c–i, where the top layer (light purple) represents the epidermal layer, and the yellow layer corresponds to the dermal layer with the channel. The morphology and cross-section of the tubular structure were examined using SEM (Fig. 7c–ii, iii). The aligned fibers in Fig. 7c–ii promoted and guided HUVEC growth and migration in specific directions, enhancing biological functions such as vascularization. The cell distribution and spreading are demonstrated in Fig. 7d–i, where spindle-shaped HUVEC cells grew along the PCL fibers after 3 days of culture. H&E staining of the cellular tubular structure (Fig. 7d–ii) revealed a well-formed HUVEC ring around the circular structure. The diameter of the tubular structure was approximately 1.2 mm, slightly smaller than the original channel diameter. The tensile modulus of the cellular vascular grafts was approximately 0.22 MPa, and the compressive modulus was around 0.17 MPa. The tensile Young's modulus and compressive modulus confirm the softness of the obtained grafts, making them suitable for blood vessel tissue engineering [109,110].

Fig. 7d–iii illustrates the integration of a cellular tubular structure within the pre-existing SE. Short-term coculture was conducted to avoid the penetration and migration of NHDF to the HUVEC layer. After 1-day coculture, the low-magnification overview (5x, Fig. 7d–iii) shows the hybrid composite structure, highlighting a clear connection between the dermal layer and the vascular graft. The CD31 expression, a biomarker for HUVEC cell junctions, along with DAPI for nuclei staining was shown in Fig. 7d–iv, which indicates the endothelial formation.

The perfusion test was performed to confirm the function of the vascular grafts within the SEs. During the medium injection process (see the video in SI), no leakage occurred, and the tubular structure retained its conformation, confirming the effective functionality of the as a blood vessel in the SEs. The efficacy of RTA in enhancing endothelial formation was evaluated. During the culture process, samples were treated with 100 μM RTA for 4 days, then samples were sectioned and stained with CD31, however, no significant difference in CD31 expression was observed following RTA treatment, as shown in the immunostaining images (Fig. SI 9).

Vessel substitution for small diameters (<6 mm) often fails due to compliance mismatch and clotting. Previous studies have produced small MEW-printed tubular PCL structures (ca. Φ 2 mm) and modified them with fibrinogen or fibrinogen/heparin coatings [70,71]. The present study achieved a much smaller diameter (ca. Φ 1 mm), and encapsulating the HUVEC/Matrigel suspension on the outer layer of the tubular structure can avoid surface modifications on scaffolds [69,71]. These findings suggest that the proposed approach can lead to fully vascularized SEs, offering the potential for wider applications *in vitro*. However, the short-term coculture is insufficient for establishing robust cell-cell interactions among keratinocytes, fibroblasts, and HUVECs, which are critical for effective skin tissue engineering [[111], [112], [113]]. The tensile modulus of the SEs with vascular grafts was approximately 2 MPa, similar to that of the SEs without vascular grafts [92], indicating that vascular integration did not compromise the tensile modulus. The compressive modulus of the SEs with vascular grafts was 106.7 ± 0.4 kPa, which also falls within the range of native skin (60–400 kPa) [92]. Further modifications and assessments to optimize this system should be involved in future work. This will be a crucial research direction for advancing vascularized skin tissue engineering, with promising applications for enhancing wound healing and opening new avenues for soft tissue engineering and repair in future studies.

## 4 Conclusion

After design optimization, a 3D skin model was developed by constructing a multilayer hybrid scaffold and culturing cells for 7 days. The biofabrication process involved sequentially casting fibroblast-laden GelMA into a two-zone PCL scaffold, printed using melt electrowriting (MEW), followed by seeding keratinocytes on top. This setup created a conducive co-cultivation environment for skin cells, resulting in mechanical properties closely resembling those of native human skin. Histological analysis and immunostaining revealed that the artificial skin model exhibited biological features comparable to human skin. Furthermore, RTA treatment enhanced ECM deposition in the SEs, validating the SEs as an effective skin model. Additionally, vascular graft integration, achieved by combining a HUVEC/Matrigel suspension with an MEW-printed tubular structure, confirmed the utility of the proposed approach in mimicking vascularized skin models. In conclusion, the versatile and reproducible SEs proposed here, present a promising prototype for future tissue testing platforms, with potential for further development through the introduction of specific cell lines or vascular structures.

## CRediT authorship contribution statement

**Xixi Wu:** Writing – review & editing, Writing – original draft, Visualization, Validation, Software, Methodology, Investigation, Formal analysis, Data curation, Conceptualization. **Fenghua Zhao:** Resources, Data curation. **Hui Wang:** Writing – review & editing, Investigation. **Romana Schirhagl:** Writing – review & editing, Supervision, Project administration, Methodology, Funding acquisition, Formal analysis. **Małgorzata K. Włodarczyk-Biegun:** Writing – review & editing, Supervision, Project administration, Methodology, Funding acquisition, Formal analysis.

## Declaration of competing interest

The authors declare that they have no known competing financial interests or personal relationships that could have appeared to influence the work reported in this paper.

## Data Availability

Data will be made available on request.
