# Supplementary material for: Integrating melt electrowriting (MEW) PCL scaffolds with fibroblast-laden hydrogel toward vascularized skin tissue engineering
Source: Mater Today Bio. 2025 Feb 19;31:101593. doi: 10.1016/j.mtbio.2025.101593 (PMC11914512; doi:10.1016/j.mtbio.2025.101593)
Supplement: Multimedia component 1 [file mmc1.docx]

Supplementary material

**Integrating Melt Electrowriting (MEW) PCL Scaffolds with Fibroblast-Laden Hydrogel toward Vascularized Skin Tissue Engineering**

Xixi Wu^a^, Fenghua Zhao^a^, Hui Wang^b^, Romana Schirhagl^a^, Małgorzata K. Włodarczyk-Biegun^c, d, *^

^a^ Department of Biomedical Engineering, University Medical Centre Groningen and University of Groningen, Ant. Deusinglaan 1, 9713 AV Groningen, The Netherlands

^b^ Nanostructured Materials and Interfaces, Zernike Institute for Advanced Materials, Faculty of Science and Engineering, University of Groningen, Nijenborgh 4, 9747 AG, the Netherlands

^c^ Polymer Science, Zernike Institute for Advanced Materials, University of Groningen, Nijenborgh 4, 9747 AG, The Netherlands

^d^ Biotechnology Centre, The Silesian University of Technology, Krzywoustego 8, 44-100 Gliwice, Poland

*Corresponding author: Małgorzata K. Włodarczyk-Biegun

E-mail: malgorzata.wlodarczyk-biegun @polsl.pl, m.k.wlodarczyk@rug.nl

Coauthors’ emails from left to right: [sissi.wu@rug.nl](mailto:sissi.wu@rug.nl), [f.zhao@umcg.nl](mailto:f.zhao@umcg.nl), hui.wang@rug.nl, [romana.schirhagl@gmail.com](mailto:romana.schirhagl@gmail.com)


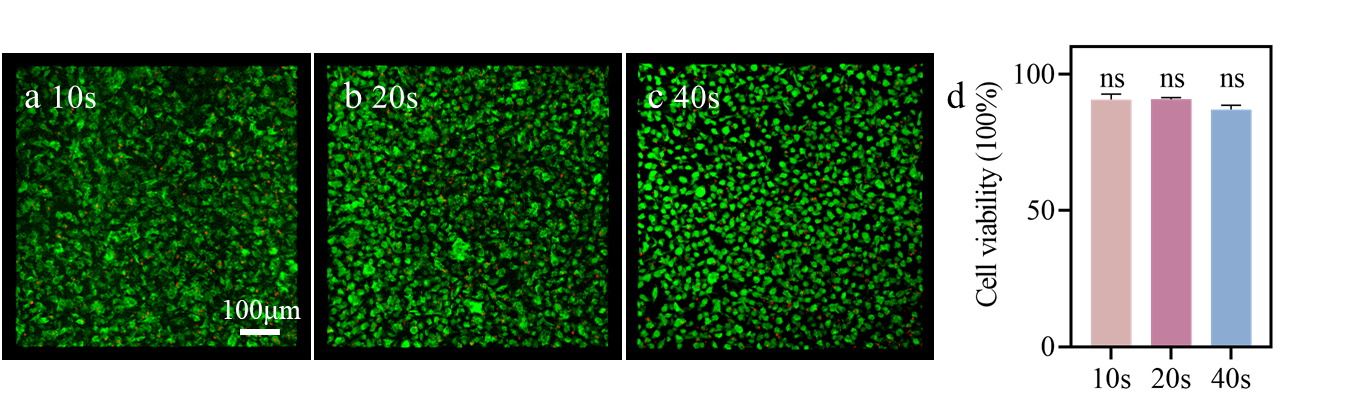


Figure S1. a) Live and dead staining of NHDF cells in 10 s, 20 s, and 40 s UV treated GelMA (5% w/v, 0.25% w/v LAP) after 7-day culture, live cells are green, dead cells are in red. b) Summary of the cell viability. The statistical significance was done using the ANOVA test, ns indicates no significant difference between the experimental groups. Scale bar: 100 μm.


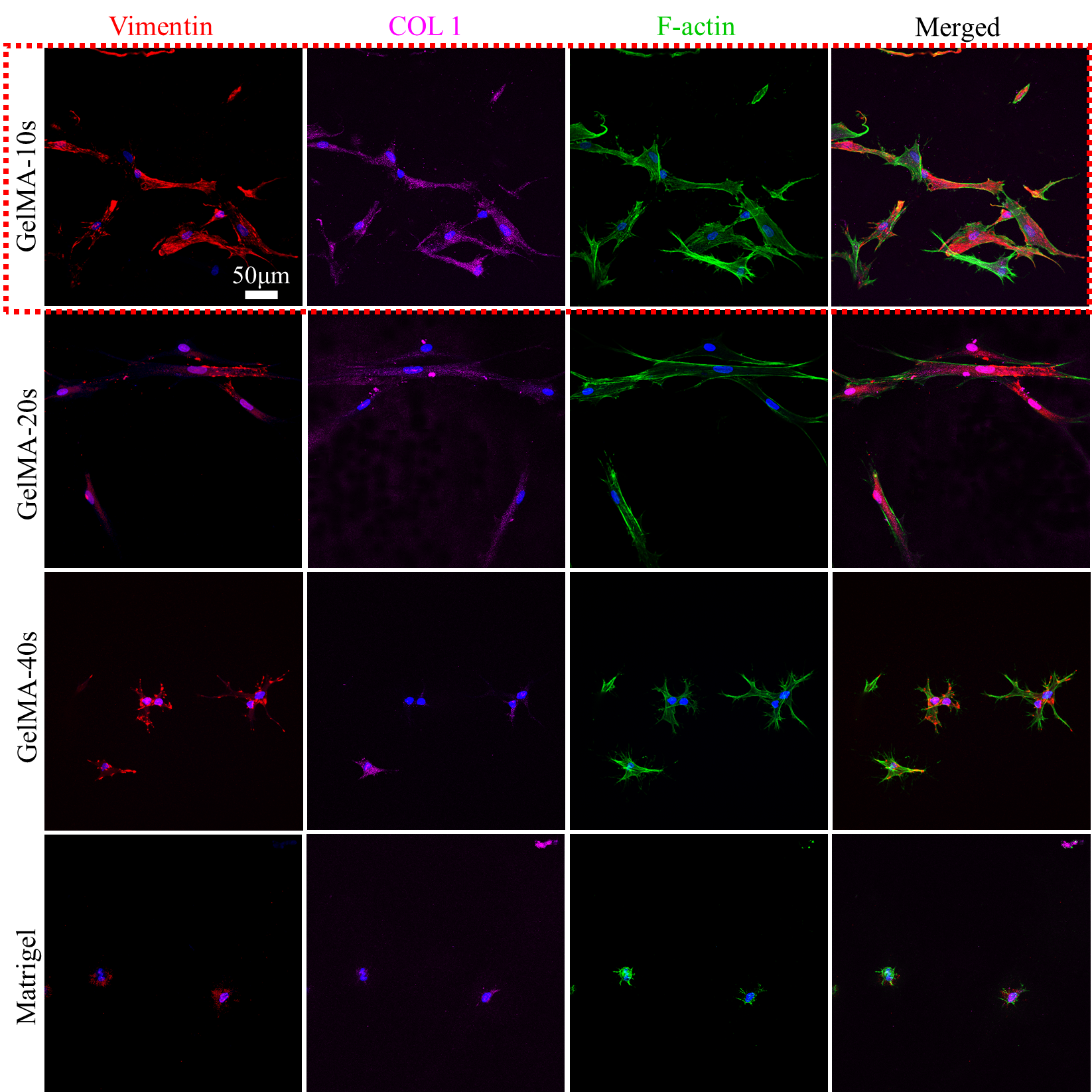
Figure S2. Cell growth in GelMA (10, 20, and 40s UV crosslinking) and Matrigel after 1-week culture. Vimentin (red), Collagen I, F-actin (green), and cell nuclei (blue) were stained to show morphology and migration capacity, Collagen I secretion, spreading, and localization of cells individually. Scale bar: 50 μm.


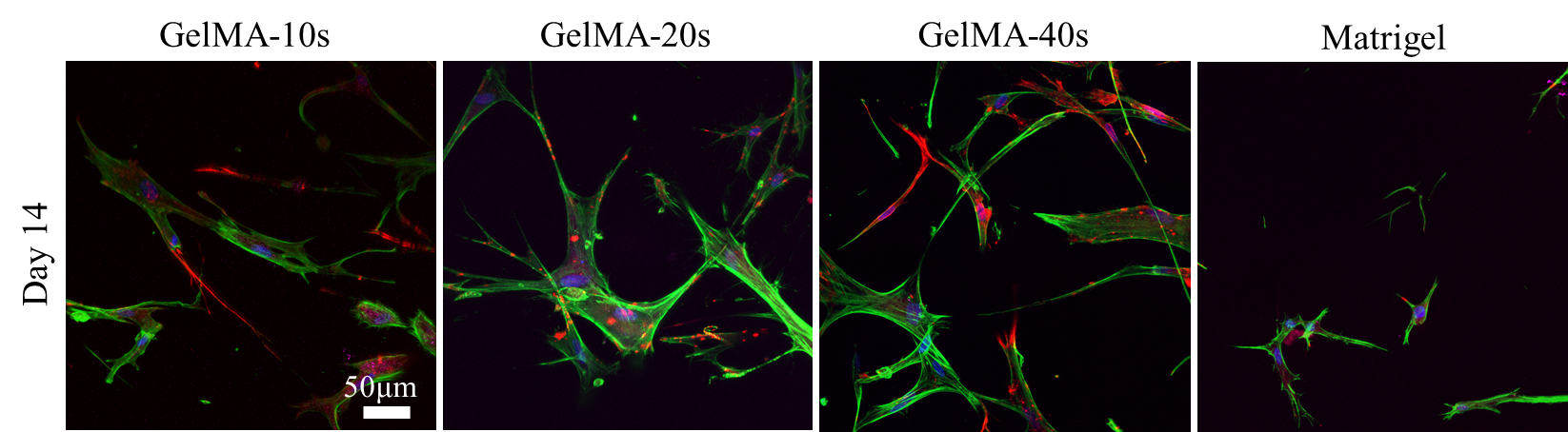
Figure S3. NHDF cell growth in gels. Spindle-shaped cells were observed clearly in Matrigel after a 14-day culture. The f-actin was in green, vimentin was labeled in red, Collagen I was marked in magenta, and the cell nuclei were in blue. 3 samples of each experimental group were assessed. 5-6 views of each sample were observed under the confocal microscope. Scale bar: 100 μm.


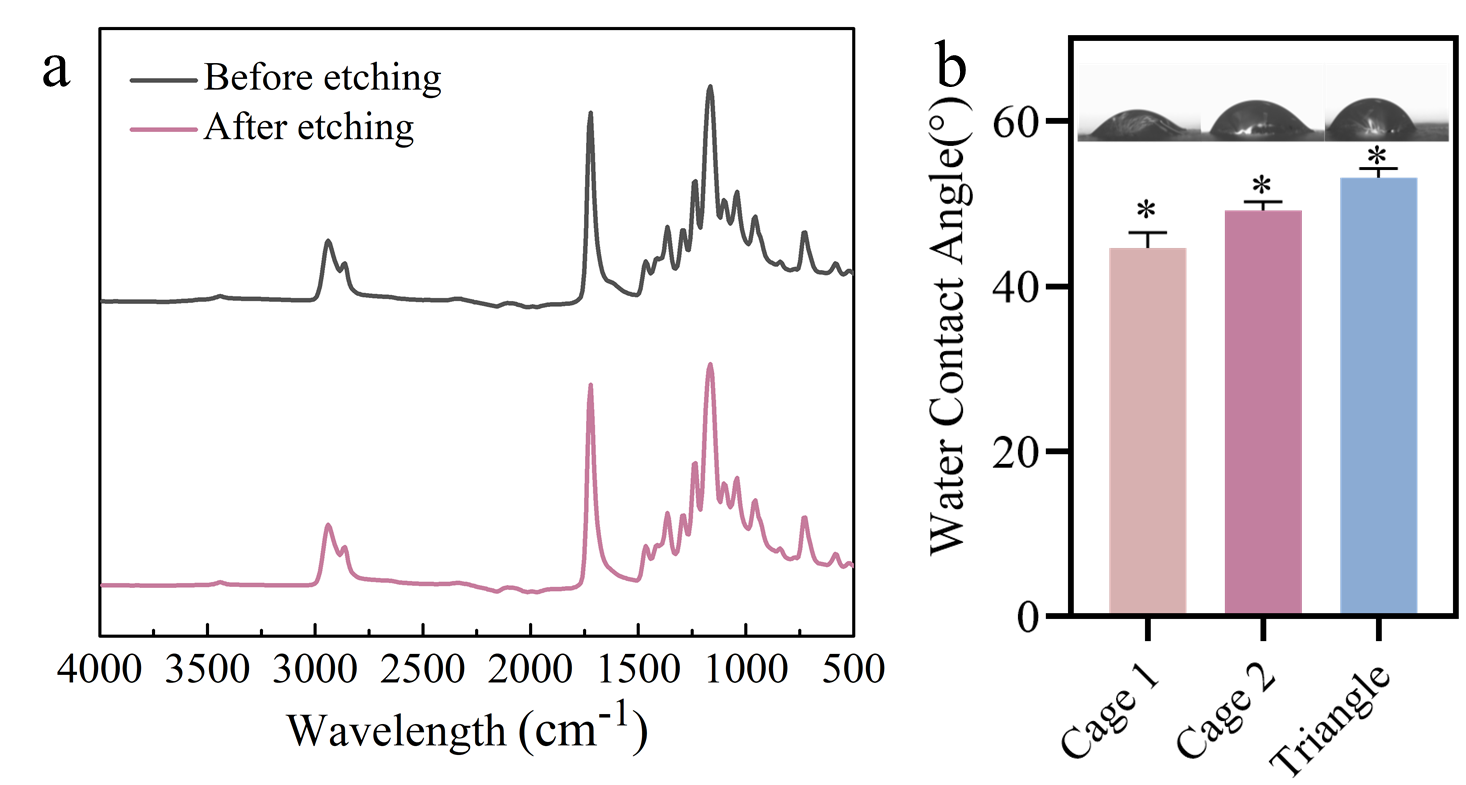


Figure S4. a) FTIR of pristine PCL scaffolds and etched PCL scaffolds. f) The water contact angle of the untreated PCL scaffolds and alkali-treated (etched) scaffolds.


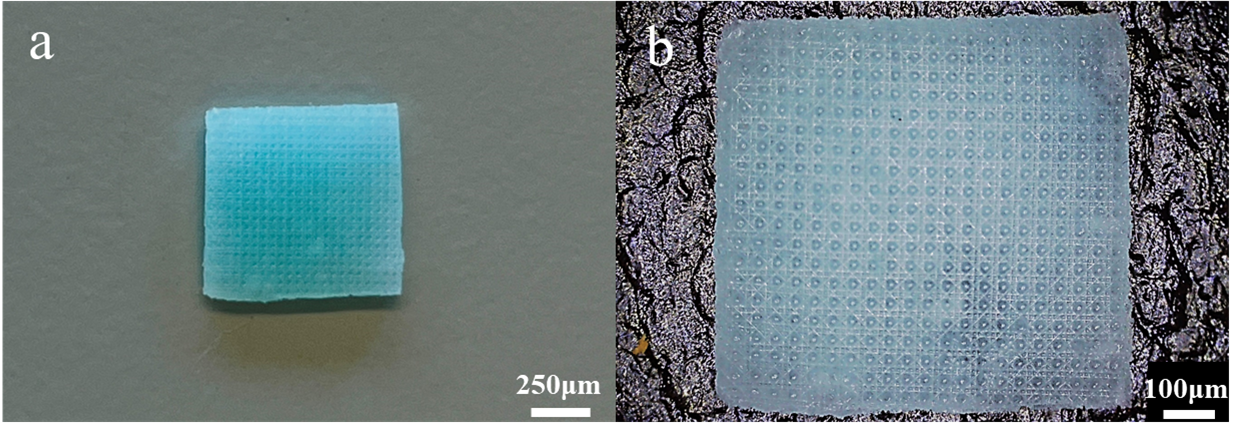


Figure S5. Optical images of PCL/GelMA composite. Scale bars: (a) 250 μm, (b) 100 μm.

Table 1: Degradation profile of PCL/GelMA composites measured by weight loss (%)


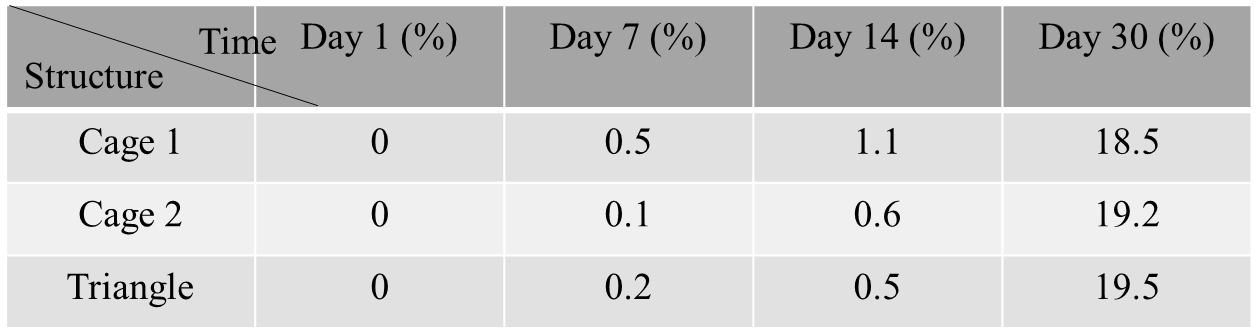


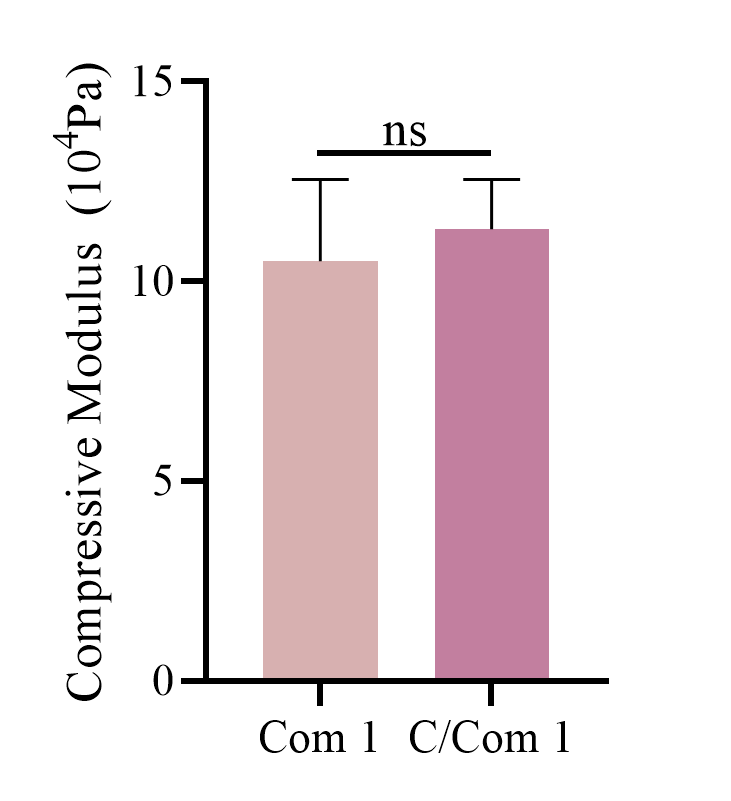


Figure S6. a) Compressive modulus of Cage 2 composite without (Com 1) and with NHDF cells (C/Com 1) at day 1. There is no significant difference shown in these two groups.


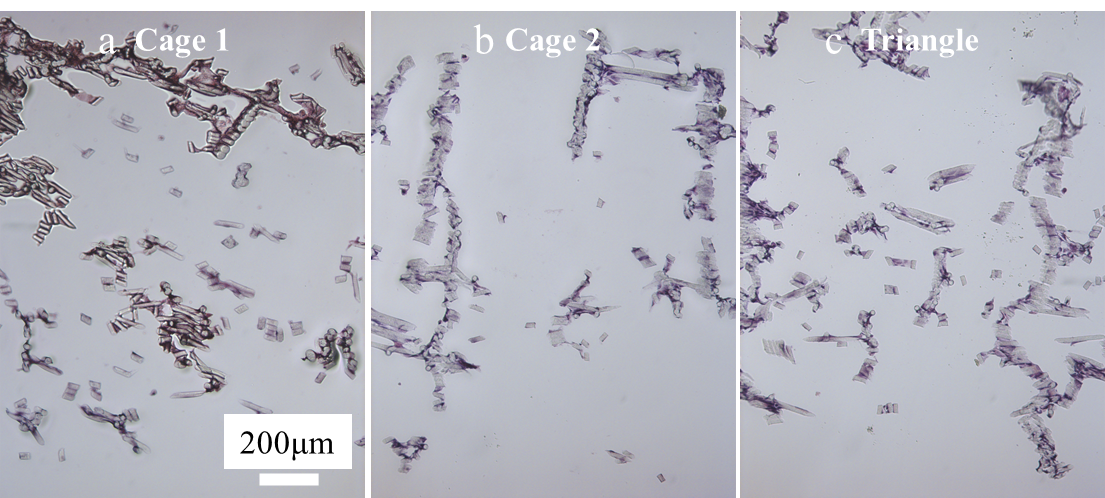


Figure S7. H&E staining of the section slices of cell-laden Cage 1, 2, and Triangle structures. Cell nuclei are purplish blue, cytoplasmic components are pink. No obvious epidermal and dermal layers were observed. Scale bar: 200 μm.


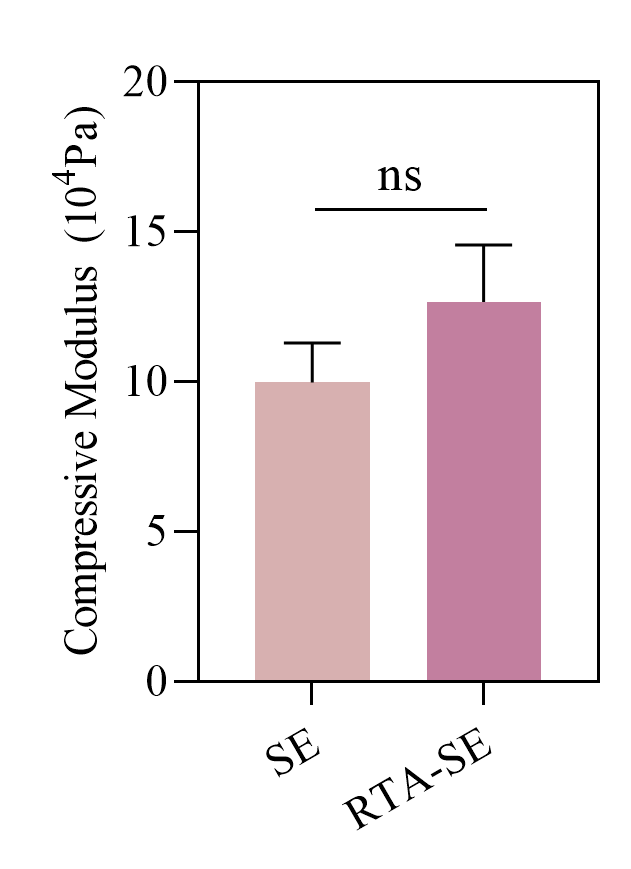


Figure S8. Compressive modulus of untreated SEs and RTA-treated SEs after 10-day culture.


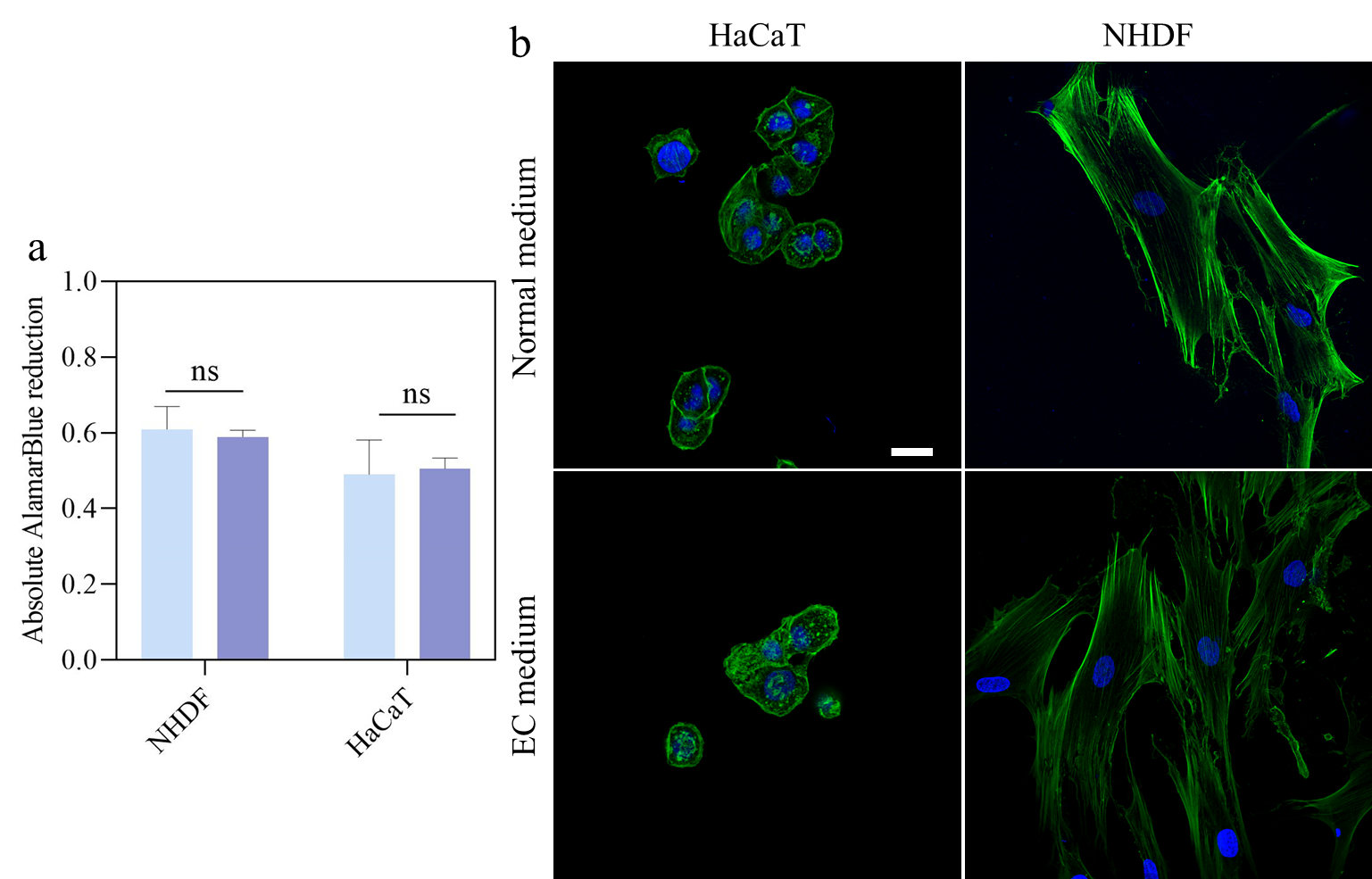


Figure S9. a) The metabolism of NHDF and HaCaT using the normal medium and EC medium after 7-day culture. b) The spreading of NHDF and HaCaT after 7-day culture in the normal (10% FBS with 90% DMEM, and , and 1% penicillin-streptomycin) and EC medium. F-actin was stained in green, and cell nuclei were stained in blue. Scale bar: 20 μm.


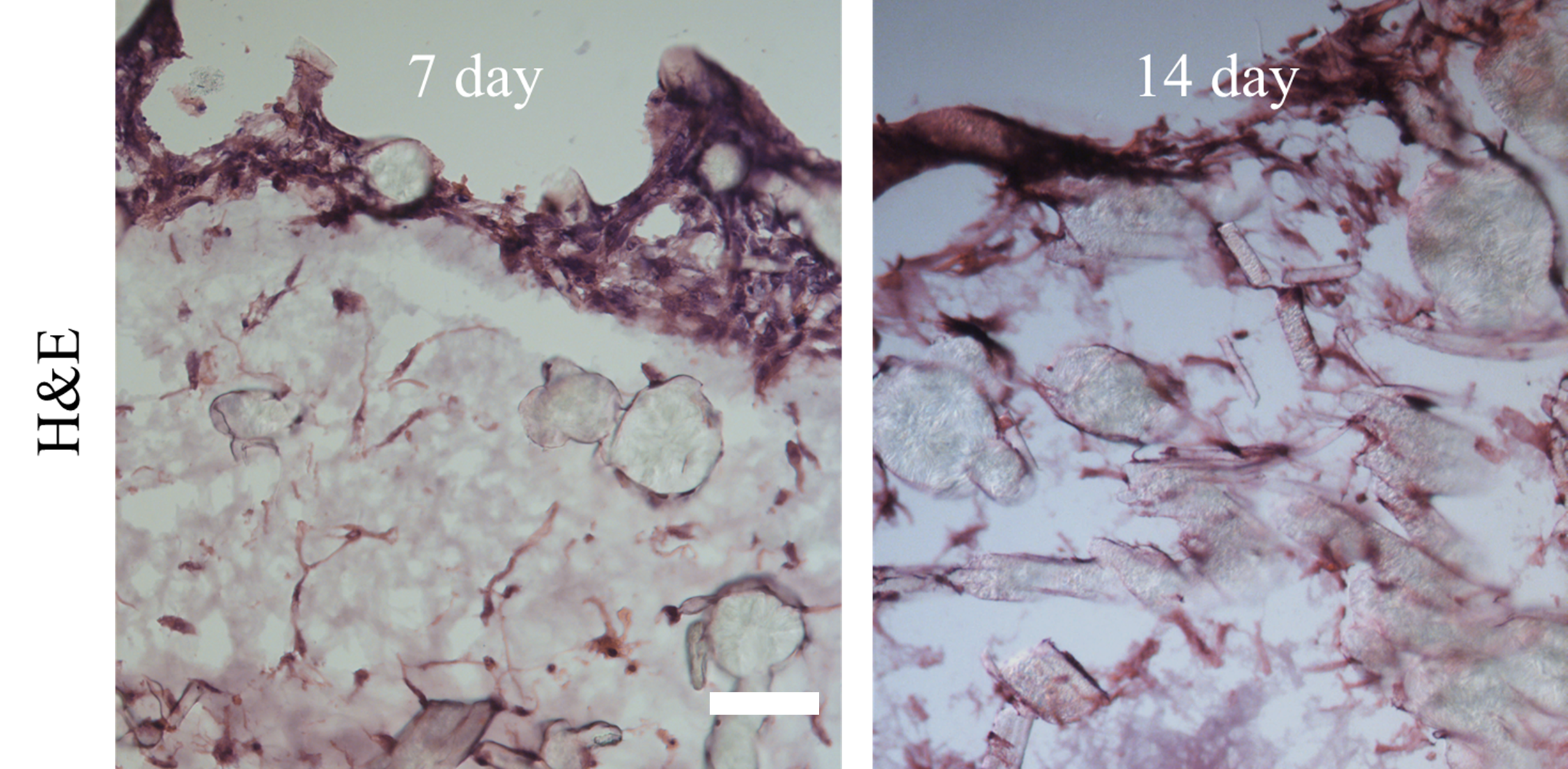


Figure S10. H&E staining of the section slices of Cage 1-SE after 7 and 14 days of culture. Cell nuclei are purplish blue, cytoplasmic components are pink. Scale bar:100 μm.


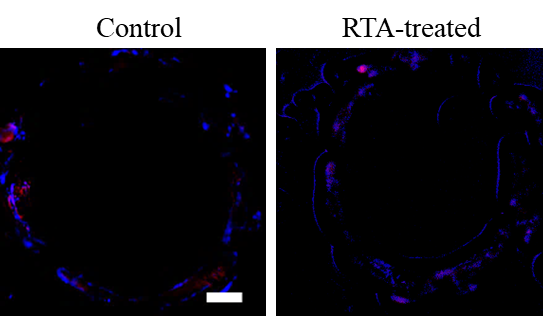


Figure S11. The expression of CD 31 of the vascular structure before and after RTA treatment. Nuclei were stained in blue, CD 31 was stained in red. The scale bar is 200 μm.
